# Supplementary material for: First historical genome of a crop bacterial pathogen from herbarium specimen: Insights into citrus canker emergence
Source: PLoS Pathog. 2021 Jul 29;17(7):e1009714. doi: 10.1371/journal.ppat.1009714 (PMC8320980; doi:10.1371/journal.ppat.1009714)
Supplement: S1 Fig — (PDF) [file ppat.1009714.s001.pdf]

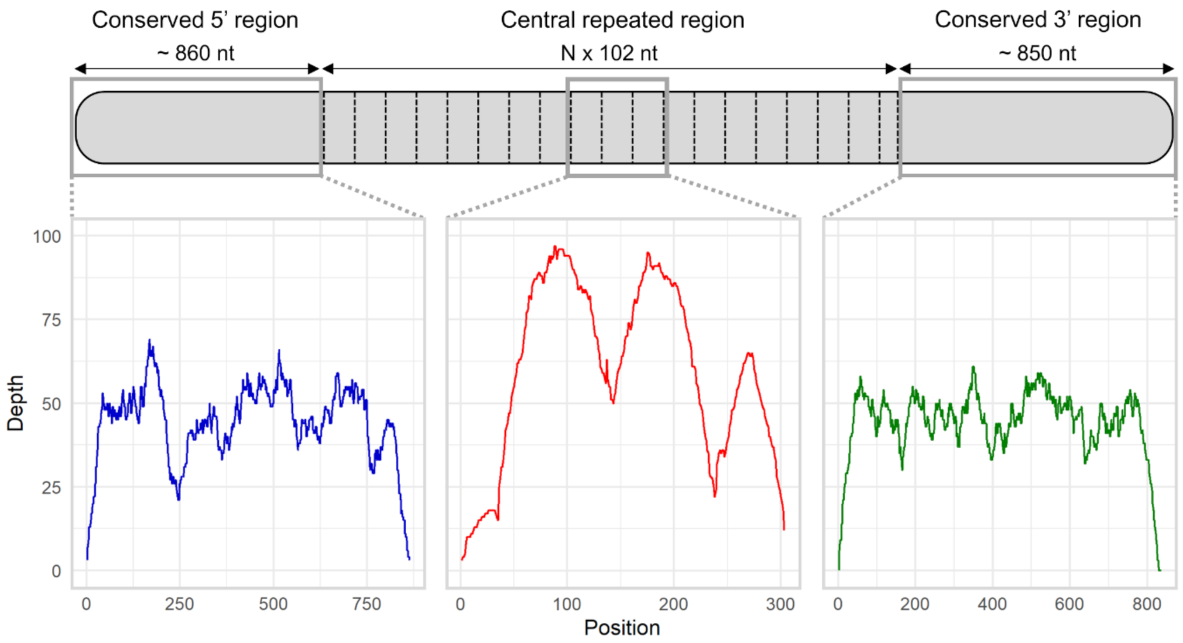


**S1 Fig. Reads depth of a Transcription Activator-Like Effector (TALE) gene of HERB_1937.**

Coverage of HERB_1937 reads along the three domains of a *tale* gene coding for an N-terminal domain (left, containing signals responsible for injection of the protein into the plant cell), a repeat domain (middle, encoding for a succession of 33-35 amino acids loop responsible for the specificity of binding to the promoters of plant target genes) and a C-terminal domain (right, containing the nuclear localization signals and transcriptional activation domain) [1].

## References


1. Boch J, Bonas U. *Xanthomonas* AvrBs3 family-type III effectors: discovery and function. Annu Rev Phytopathol. 2010;48:419–36. doi:10.1146/annurev-phyto-080508-081936
