# Supplementary material for: First historical genome of a crop bacterial pathogen from herbarium specimen: Insights into citrus canker emergence
Source: PLoS Pathog. 2021 Jul 29;17(7):e1009714. doi: 10.1371/journal.ppat.1009714 (PMC8320980; doi:10.1371/journal.ppat.1009714)
Supplement: S2 Fig — (PDF) [file ppat.1009714.s002.pdf]

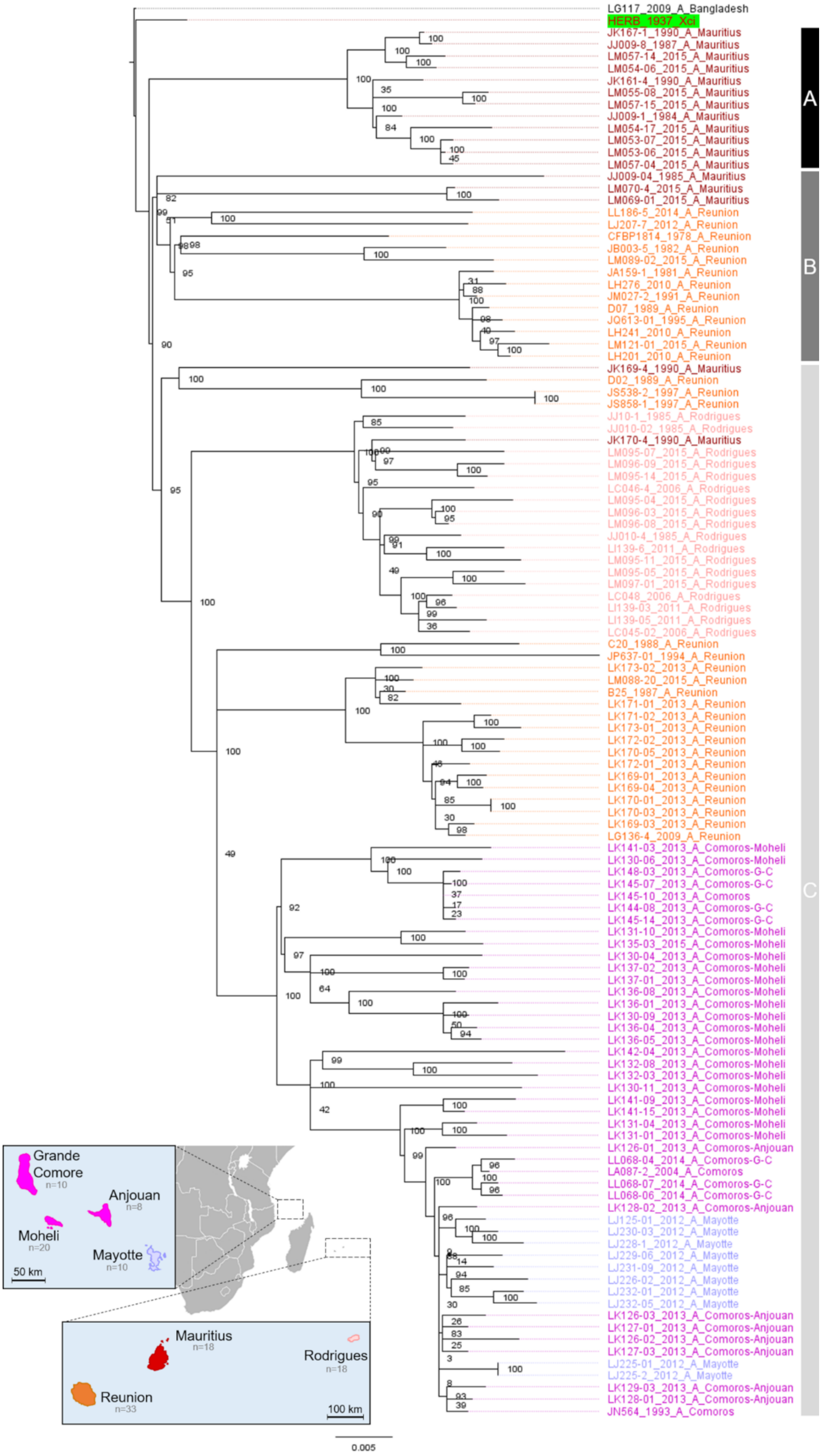
LG117_2009_A_Bangladesh
HERB_1937_Xci
JK167-1_1990_A_Mauritius
JJ009-8_1987_A_Mauritius
LM057-14_2015_A_Mauritius
LM054-06_2015_A_Mauritius
JK161-4_1990_A_Mauritius
LM055-08_2015_A_Mauritius
LM057-15_2015_A_Mauritius
JJ009-1_1984_A_Mauritius
LM054-17_2015_A_Mauritius
LM053-07_2015_A_Mauritius
LM053-06_2015_A_Mauritius
LM057-04_2015_A_Mauritius
JJ009-04_1985_A_Mauritius
LM070-4_2015_A_Mauritius
LM069-01_2015_A_Mauritius
LL186-5_2014_A_Reunion
LJ207-7_2012_A_Reunion
CFBP1814_1978_A_Reunion
JB003-5_1982_A_Reunion
LM089-02_2015_A_Reunion
JA159-1_1981_A_Reunion
LH276_2010_A_Reunion
JM027-2_1991_A_Reunion
D07_1989_A_Reunion
JQ613-01_1995_A_Reunion
LH241_2010_A_Reunion
LM121-01_2015_A_Reunion
LH201_2010_A_Reunion
JK169-4_1990_A_Mauritius
D02_1989_A_Reunion
JS538-2_1997_A_Reunion
JS858-1_1997_A_Reunion
JJ10-1_1985_A_Rodrigues
JJ010-02_1985_A_Rodrigues
JK170-4_1990_A_Mauritius
LM095-07_2015_A_Rodrigues
LM096-09_2015_A_Rodrigues
LM095-14_2015_A_Rodrigues
LC046-4_2006_A_Rodrigues
LM095-04_2015_A_Rodrigues
LM096-03_2015_A_Rodrigues
LM096-08_2015_A_Rodrigues
JJ010-4_1985_A_Rodrigues
LI139-6_2011_A_Rodrigues
LM095-11_2015_A_Rodrigues
LM095-05_2015_A_Rodrigues
LM097-01_2015_A_Rodrigues
LC048_2006_A_Rodrigues
LI139-03_2011_A_Rodrigues
LI139-05_2011_A_Rodrigues
LC045-02_2006_A_Rodrigues
C20_1988_A_Reunion
JP637-01_1994_A_Reunion
LK173-02_2013_A_Reunion
LM088-20_2015_A_Reunion
B25_1987_A_Reunion
LK171-01_2013_A_Reunion
LK171-02_2013_A_Reunion
LK173-01_2013_A_Reunion
LK172-02_2013_A_Reunion
LK170-05_2013_A_Reunion
LK172-01_2013_A_Reunion
LK169-01_2013_A_Reunion
LK169-04_2013_A_Reunion
LK170-01_2013_A_Reunion
LK170-03_2013_A_Reunion
LK169-03_2013_A_Reunion
LG136-4_2009_A_Reunion
LK141-03_2013_A_Comoros-Moheli
LK130-06_2013_A_Comoros-Moheli
LK148-03_2013_A_Comoros-G-C
LK145-07_2013_A_Comoros-G-C
LK145-10_2013_A_Comoros
LK144-08_2013_A_Comoros-G-C
LK145-14_2013_A_Comoros-G-C
LK131-10_2013_A_Comoros-Moheli
LK135-03_2015_A_Comoros-Moheli
LK130-04_2013_A_Comoros-Moheli
LK137-02_2013_A_Comoros-Moheli
LK137-01_2013_A_Comoros-Moheli
LK136-08_2013_A_Comoros-Moheli
LK136-01_2013_A_Comoros-Moheli
LK130-09_2013_A_Comoros-Moheli
LK136-04_2013_A_Comoros-Moheli
LK136-05_2013_A_Comoros-Moheli
LK142-04_2013_A_Comoros-Moheli
LK132-08_2013_A_Comoros-Moheli
LK132-03_2013_A_Comoros-Moheli
LK130-11_2013_A_Comoros-Moheli
LK141-09_2013_A_Comoros-Moheli
LK141-15_2013_A_Comoros-Moheli
LK131-04_2013_A_Comoros-Moheli
LK131-01_2013_A_Comoros-Moheli
LK126-01_2013_A_Comoros-Anjouan
LL068-04_2014_A_Comoros-G-C
LA087-2_2004_A_Comoros
LL068-07_2014_A_Comoros-G-C
LL068-06_2014_A_Comoros-G-C
LK128-02_2013_A_Comoros-Anjouan
LJ125-01_2012_A_Mayotte
LJ230-03_2012_A_Mayotte
LJ228-1_2012_A_Mayotte
LJ229-06_2012_A_Mayotte
LJ231-09_2012_A_Mayotte
LJ226-02_2012_A_Mayotte
LJ232-01_2012_A_Mayotte
LJ232-05_2012_A_Mayotte
LK126-03_2013_A_Comoros-Anjouan
LK127-01_2013_A_Comoros-Anjouan
LK126-02_2013_A_Comoros-Anjouan
LK127-03_2013_A_Comoros-Anjouan
LJ225-01_2012_A_Mayotte
LJ225-2_2012_A_Mayotte
LK129-03_2013_A_Comoros-Anjouan
LK128-01_2013_A_Comoros-Anjouan
JN564_1993_A_Comoros
A
B
C
Grande Comore
n=10
Anjouan
n=8
Moheli
n=20
Mayotte
n=10
50 km
Mauritius
n=18
Rodrigues
n=18
Reunion
n=33
100 km
0.005

S2 Fig. Maximum Likelihood (ML) phylogenetic tree of *Xci* genomes.

ML tree of historical HERB_1937_Xci and 116 modern strains sampled from the SWIO islands built from 2,632 chromosomal non-recombining SNPs. Bangladesh strain LG117 isolated in 2009 (GenBank accession number: CDAX01000000) was used as outgroup. HERB_1937_Xci genome is highlighted in green. Node values correspond to bootstrap values calculated on 1,000 iterations. Tip labels indicate sample reference ID, date of collection, pathotype and locality; colors differ according to the geographic origin of the sample. The tree is structured in three lineages specified by the right-hand side greyscale: Mauritius lineage (A), strains from Mauritius & Reunion (B), and strains from all SWIO islands (C). Map layer is from Natural Earth, available from https://www.naturalearthdata.com. SWIO: South West Indian Ocean, Comoros-G-C: Comoros Grande Comore.
