# Supplementary material for: First historical genome of a crop bacterial pathogen from herbarium specimen: Insights into citrus canker emergence
Source: PLoS Pathog. 2021 Jul 29;17(7):e1009714. doi: 10.1371/journal.ppat.1009714 (PMC8320980; doi:10.1371/journal.ppat.1009714)
Supplement: S3 Fig — (PDF) [file ppat.1009714.s003.pdf]

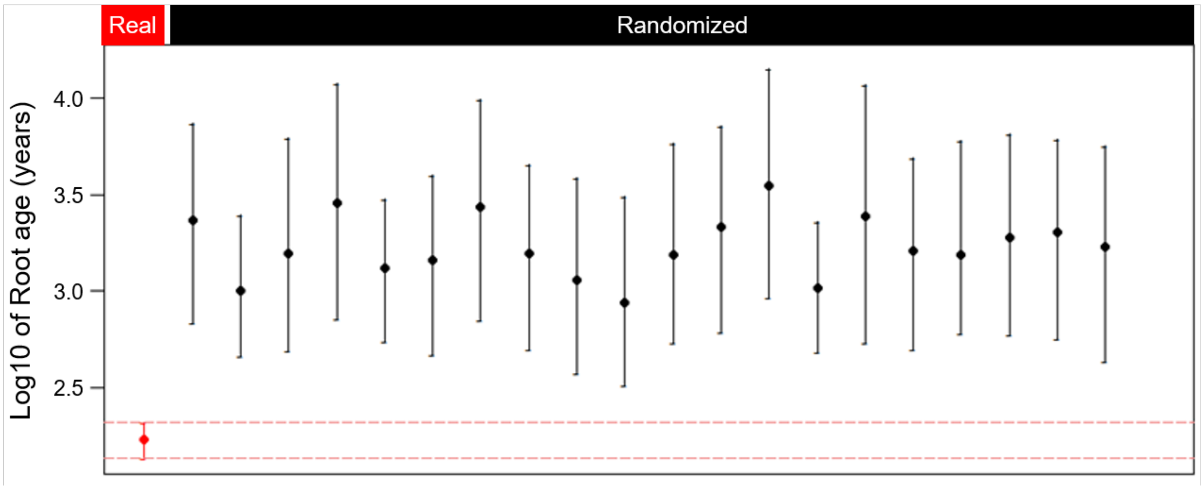

S3 Fig. Date-randomization test results.

Evaluating temporal signal in the dataset by date-randomization test showed no overlap between the age of the root estimated from the real dataset (red) *vs* 20 date-randomized datasets (black). Vertical bars represent 95% Highest Posterior Density intervals.
