# Supplementary material for: First historical genome of a crop bacterial pathogen from herbarium specimen: Insights into citrus canker emergence
Source: PLoS Pathog. 2021 Jul 29;17(7):e1009714. doi: 10.1371/journal.ppat.1009714 (PMC8320980; doi:10.1371/journal.ppat.1009714)
Supplement: S4 Fig — (PDF) [file ppat.1009714.s004.pdf]

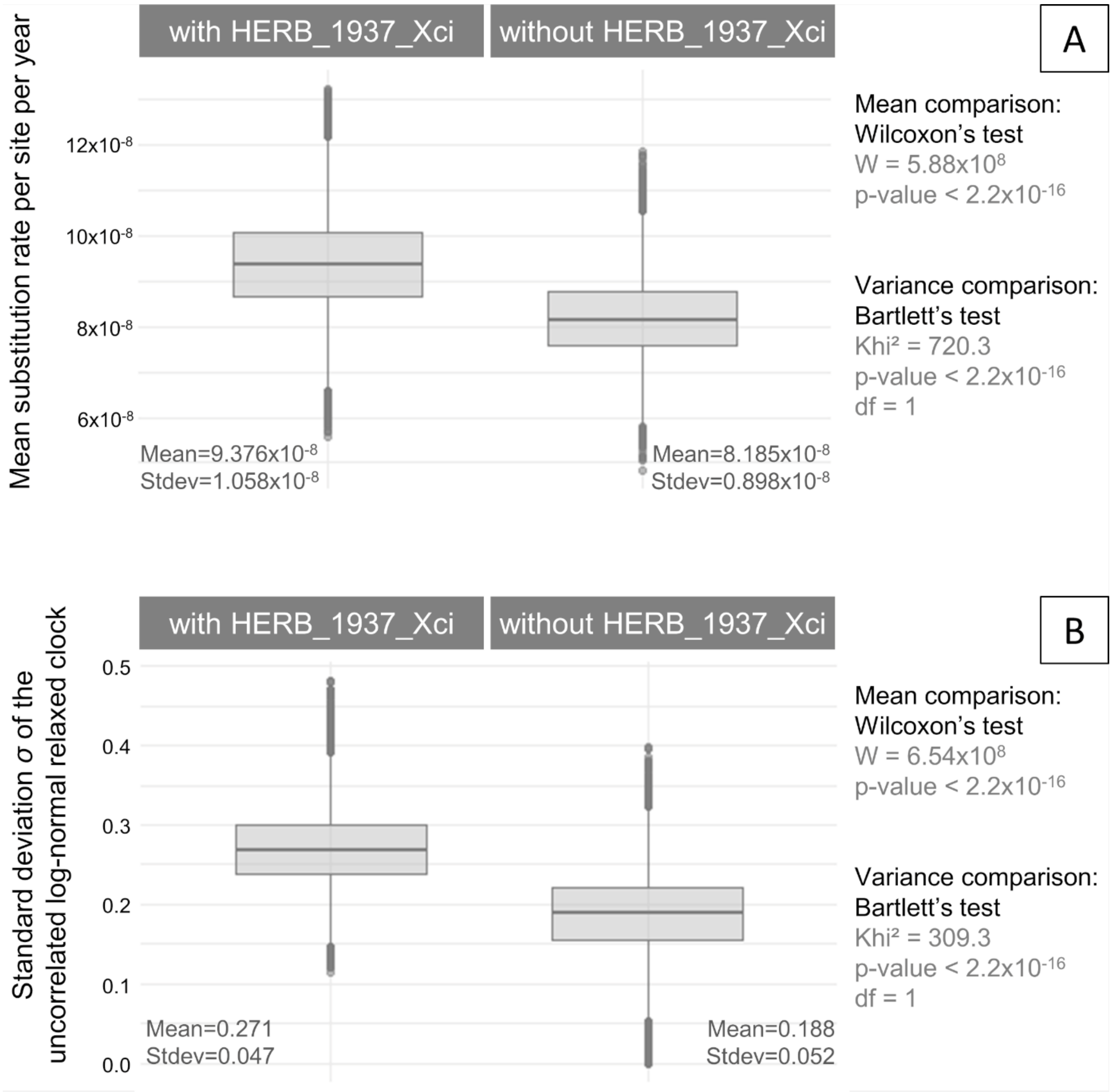

S4 Fig. Effect of integrating HERB_1937_Xci on substitution rate estimates in BEAST.

Boxplot distribution of mean substitution rate per site per year (A) and standard deviation of the uncorrelated log-normal relaxed clock among dated BEAST tree branches (B), with and without integrating the historical HERB_1937_Xci chromosome sequence in the BEAST analysis, and associated statistical comparisons. Boxes represent 25th to 75th percentiles, Minimum-Maximum intervals are displayed by a vertical bar and outliers as circles.
