# Supplementary material for: First historical genome of a crop bacterial pathogen from herbarium specimen: Insights into citrus canker emergence
Source: PLoS Pathog. 2021 Jul 29;17(7):e1009714. doi: 10.1371/journal.ppat.1009714 (PMC8320980; doi:10.1371/journal.ppat.1009714)
Supplement: S1 Table — (PDF) [file ppat.1009714.s005.pdf]

S1 Table. Published modern genomes included in the phylogenetic analyzes.

Isolate	Host	Country	Date	GenBank accession number	Sequence Read Archive accession no. (Illumina library no. 1)	Publication
B25	*Citrus hystrix*	Reunion	1987	JAABHM000000000	SRR11234853	[1]
C20	*Citrus* x *paradisi*	Reunion	1988	JAABHN000000000	SRR11234852	[1]
CFBP1814	*Citrus maxima*	Reunion	1978	JAABHO000000000	SRR11234630	[1]
D02	*Citrus* x *tangelo*	Reunion	1989	JAABHP000000000	SRR11234840	[1]
D07	*Citrus* x *unshiu*	Reunion	1989	JAABHQ000000000	SRR11234829	[1]
JA159-1	*Citrus* x *clementina*	Reunion	1981	JAABHR000000000	SRR11234796	[1]
JB003-5	*Citrus* x *aurantiifolia*	Reunion	1982	JAABHS000000000	SRR11234785	[1]
JJ009-04	*Citrus* sp. x *bergamia*	Mauritius	1985	JAABBT000000000	SRR11234774	[1]
JJ009-1	*Poncirus trifoliata* x *Citrus* x *sinensis*	Mauritius	1984	JAABBU000000000	SRR11234763	[1]
JJ009-8	*Citrus* x *sinensis*	Mauritius	1987	JAABBV000000000	SRR11234752	[1]
JJ010-02	*Citrus* x *aurantiifolia*	Rodrigues	1985	JAABHW000000000	SRR11234740	[1]
JJ010-4	*Citrus* x *aurantiifolia*	Rodrigues	1985	JAABHX000000000	SRR11234729	[1]
JJ10-1	*Citrus* x *aurantiifolia*	Rodrigues	1985	CDDV01000000	SRR11234658	[2]
JK161-4	*Citrus* x *sinensis*	Mauritius	1990	JAABBW000000000	SRR11234619	[1]
JK167-1	*Citrus* x *aurantiifolia*	Mauritius	1990	JAABBX000000000	SRR11234618	[1]
JK169-4	*Citrus* sp.	Mauritius	1990	JAABBY000000000	SRR11234617	[1]
JK170-4	*Citrus* x *aurantiifolia*	Mauritius	1990	JAABBZ000000000	SRR11234616	[1]
JM027-2	*Citrus* x *limon*	Reunion	1991	JAABHT000000000	SRR11234615	[1]
JN564	*Citrus* sp.	Comoros (Grande Comore)	1993	JAAAZK000000000	SRR11234613	[1]
JP637-01	*Citrus reticulata*	Reunion	1994	JAABHL000000000	SRR11234610	[1]
JQ613-01	*Citrus hystrix*	Reunion	1995	JAABGQ000000000	SRR11234602	[1]
JS538-2	*Citrus* x *paradisi*	Reunion	1996	JAABHE000000000	SRR11234598	[1]
JS858-1	*Citrus* x *paradisi*	Reunion	1996	JAABHF000000000	SRR11234597	[1]
LA087-2	*Citrus* sp.	Comoros (Grande Comore)	2004	JAAAZL000000000	SRR11234593	[1]
LC045-02	*Citrus* x *aurantiifolia*	Rodrigues	2006	JAABHZ000000000	SRR11234589	[1]

LC046-4	*Citrus* x *aurantiifolia*	Rodrigues	2006	JAABIA000000000	SRR11234588	[1]
LC048	*Citrus* x *aurantiifolia*	Rodrigues	2006	JAABIB000000000	SRR11234587	[1]
LG117	*Citrus* sp.	Bangladesh	2009	CDAX01000000	SRR11234648	[2]
LG136-4	*Citrus hystrix*	Reunion	2009	JAABGM000000000	SRR11234585	[1]
LH201	*Citrus hystrix*	Reunion	2010	JAABEJ000000000	SRR11234584	[1]
LH241	*Citrus hystrix*	Reunion	2010	JAABFJ000000000	SRR11234578	[1]
LH276	*Citrus reticulata* x *sinensis*	Reunion	2010	JAABHU000000000	SRR11234577	[1]
LI139-03	*Citrus* x *aurantiifolia*	Rodrigues	2011	JAABIC000000000	SRR11234569	[1]
LI139-05	*Citrus* x *aurantiifolia*	Rodrigues	2011	JAABID000000000	SRR11234568	[1]
LI139-6	*Citrus* x *aurantiifolia*	Rodrigues	2011	JAABIE000000000	SRR11234567	[1]
LJ125-01	*Citrus* x *sinensis*	Mayotte	2012	JAABCK000000000	SRR11234555	[1]
LJ207-7	*Citrus hystrix*	Reunion	2012	JAABEI000000000	SRR11234554	[1]
LJ225-01	*Citrus* x *sinensis*	Mayotte	2012	JAABCL000000000	SRR11234553	[1]
LJ225-2	*Citrus* x *sinensis*	Mayotte	2012	JAABCM000000000	SRR11234552	[1]
LJ226-02	*Citrus* x *sinensis*	Mayotte	2012	JAABCN000000000	SRR11234551	[1]
LJ228-1	*Citrus hystrix*	Mayotte	2012	JAABCO000000000	SRR11234855	[1]
LJ229-06	*Citrus reticulata*	Mayotte	2012	JAABCP000000000	SRR11234854	[1]
LJ230-03	*Citrus* x *sinensis*	Mayotte	2012	JAABCQ000000000	SRR11234850	[1]
LJ231-09	*Citrus* sp.	Mayotte	2012	JAABCR000000000	SRR11234849	[1]
LJ232-01	*Citrus* x *sinensis*	Mayotte	2012	JAABCS000000000	SRR11234848	[1]
LJ232-05	*Citrus* x *sinensis*	Mayotte	2012	JAABCT000000000	SRR11234847	[1]
LK126-01	*Citrus* x *aurantiifolia*	Comoros (Anjouan)	2013	JAAAZC000000000	SRR11234846	[1]
LK126-02	*Citrus* x *aurantiifolia*	Comoros (Anjouan)	2013	JAAAZD000000000	SRR11234845	[1]
LK126-03	*Citrus* x *aurantiifolia*	Comoros (Anjouan)	2013	JAAAZE000000000	SRR11234844	[1]
LK127-01	*Citrus* x *sinensis*	Comoros (Anjouan)	2013	JAAAZF000000000	SRR11234843	[1]
LK127-03	*Citrus* x *sinensis*	Comoros (Anjouan)	2013	JAAAZG000000000	SRR11234842	[1]
LK128-01	*Citrus* x *sinensis*	Comoros (Anjouan)	2013	JAAAZH000000000	SRR11234841	[1]
LK128-02	*Citrus* x *sinensis*	Comoros (Anjouan)	2013	JAAAZI000000000	SRR11234839	[1]
LK129-03	*Citrus* x *aurantiifolia*	Comoros (Anjouan)	2013	JAAAZJ000000000	SRR11234838	[1]
LK130-04	*Citrus* x *aurantiifolia*	Comoros (Moheli)	2013	JAAAZU000000000	SRR11234837	[1]

LK130-06	*Citrus* x *aurantiifolia*	Comoros (Moheli)	2013	JAAAZV000000000	SRR11234836	[1]
LK130-09	*Citrus* x *aurantiifolia*	Comoros (Moheli)	2013	JAAAZW000000000	SRR11234835	[1]
LK130-11	*Citrus* x *aurantiifolia*	Comoros (Moheli)	2013	JAAAZX000000000	SRR11234834	[1]
LK131-01	*Citrus* x *sinensis*	Comoros (Moheli)	2013	JAAAZY000000000	SRR11234833	[1]
LK131-04	*Citrus* x *aurantiifolia*	Comoros (Moheli)	2013	JAAAZZ000000000	SRR11234832	[1]
LK131-10	*Citrus* x *aurantiifolia*	Comoros (Moheli)	2013	JAABAA000000000	SRR11234831	[1]
LK132-03	*Citrus* x *sinensis*	Comoros (Moheli)	2013	JAABAB000000000	SRR11234830	[1]
LK132-08	*Citrus* x *aurantiifolia*	Comoros (Moheli)	2013	JAABAC000000000	SRR11234828	[1]
LK135-03	*Citrus* sp.	Comoros (Moheli)	2015	JAABAD000000000	SRR11234827	[1]
LK136-01	*Citrus* x *aurantiifolia*	Comoros (Moheli)	2013	JAABAE000000000	SRR11234826	[1]
LK136-04	*Citrus* x *aurantiifolia*	Comoros (Moheli)	2013	JAABAF000000000	SRR11234825	[1]
LK136-05	*Citrus* x *aurantiifolia*	Comoros (Moheli)	2013	JAABAG000000000	SRR11234824	[1]
LK136-08	*Citrus* x *aurantiifolia*	Comoros (Moheli)	2013	JAABAH000000000	SRR11234823	[1]
LK137-01	*Citrus* x *sinensis*	Comoros (Moheli)	2013	JAABAI000000000	SRR11234822	[1]
LK137-02	*Citrus* x *aurantiifolia*	Comoros (Moheli)	2013	JAABAJ000000000	SRR11234821	[1]
LK141-03	*Citrus* x *sinensis*	Comoros (Moheli)	2013	JAABAK000000000	SRR11234820	[1]
LK141-09	*Citrus* x *sinensis*	Comoros (Moheli)	2013	JAABAL000000000	SRR11234819	[1]
LK141-15	*Citrus* x *sinensis*	Comoros (Moheli)	2013	JAABAM000000000	SRR11234817	[1]
LK142-04	*Citrus* x *aurantiifolia*	Comoros (Moheli)	2013	JAABAN000000000	SRR11234816	[1]
LK144-08	*Citrus* sp.	Comoros (Grande Comore)	2013	JAAAZM000000000	SRR11234815	[1]
LK145-07	*Citrus* sp.	Comoros (Grande Comore)	2013	JAAAZN000000000	SRR11234814	[1]
LK145-10	*Citrus* sp.	Comoros (Grande Comore)	2013	JAAAZO000000000	SRR11234813	[1]
LK145-14	*Citrus* sp.	Comoros (Grande Comore)	2013	JAAAZP000000000	SRR11234812	[1]
LK148-03	*Citrus* sp.	Comoros (Grande Comore)	2013	JAAAZQ000000000	SRR11234811	[1]
LK169-01	*Citrus hystrix*	Reunion	2013	JAABFW000000000	SRR11234810	[1]
LK169-03	*Citrus hystrix*	Reunion	2013	JAABFX000000000	SRR11234809	[1]
LK169-04	*Citrus hystrix*	Reunion	2013	JAABFY000000000	SRR11234808	[1]

LK170-01	*Citrus hystrix*	Reunion	2013	JAABFZ000000000	SRR11234806	[1]
LK170-03	*Citrus hystrix*	Reunion	2013	JAABGA000000000	SRR11234805	[1]
LK170-05	*Citrus hystrix*	Reunion	2013	JAABGB000000000	SRR11234804	[1]
LK171-01	*Citrus hystrix*	Reunion	2013	JAABGC000000000	SRR11234803	[1]
LK171-02	*Citrus hystrix*	Reunion	2013	JAABGD000000000	SRR11234802	[1]
LK172-01	*Citrus hystrix*	Reunion	2013	JAABGE000000000	SRR11234801	[1]
LK172-02	*Citrus hystrix*	Reunion	2013	JAABGF000000000	SRR11234800	[1]
LK173-01	*Citrus hystrix*	Reunion	2013	JAABGG000000000	SRR11234799	[1]
LK173-02	*Citrus hystrix*	Reunion	2013	JAABGH000000000	SRR11234798	[1]
LL068-04	*Citrus* sp.	Comoros (Grande Comore)	2014	JAAAZR000000000	SRR11234797	[1]
LL068-06	*Citrus* sp.	Comoros (Grande Comore)	2014	JAAAZS000000000	SRR11234795	[1]
LL068-07	*Citrus* sp.	Comoros (Grande Comore)	2014	JAAAZT000000000	SRR11234794	[1]
LL186-5	*Citrus* x *sinensis*	Reunion	2014	JAABHV000000000	SRR11234753	[1]
LM053-06	*Citrus* x *aurantiifolia*	Mauritius	2015	JAABCA000000000	SRR11234751	[1]
LM053-07	*Citrus* x *aurantiifolia*	Mauritius	2015	JAABCB000000000	SRR11234750	[1]
LM054-06	*Citrus* x *sinensis*	Mauritius	2015	JAABCC000000000	SRR11234749	[1]
LM054-17	*Citrus* x *sinensis*	Mauritius	2015	JAABCD000000000	SRR11234748	[1]
LM055-08	*Citrus reticulata* x *paradisi*	Mauritius	2015	JAABCE000000000	SRR11234747	[1]
LM057-04	*Citrus* x *meyeri*	Mauritius	2015	JAABCF000000000	SRR11234746	[1]
LM057-14	*Citrus* x *meyeri*	Mauritius	2015	JAABCG000000000	SRR11234745	[1]
LM057-15	*Citrus* x *meyeri*	Mauritius	2015	JAABCH000000000	SRR11234744	[1]
LM069-01	*Citrus* x *aurantiifolia*	Mauritius	2015	JAABCI000000000	SRR11234743	[1]
LM070-4	*Citrus* x *aurantiifolia*	Mauritius	2015	JAABCJ000000000	SRR11234742	[1]
LM088-20	*Citrus hystrix*	Reunion	2015	JAABEN000000000	SRR11234738	[1]
LM089-02	*Citrus reticulata* x *sinensis*	Reunion	2015	JAABDJ000000000	SRR11234732	[1]
LM095-04	*Citrus* x *aurantiifolia*	Rodrigues	2015	JAABIF000000000	SRR11234725	[1]
LM095-05	*Citrus* x *aurantiifolia*	Rodrigues	2015	JAABIG000000000	SRR11234724	[1]
LM095-07	*Citrus* x *aurantiifolia*	Rodrigues	2015	JAABIH000000000	SRR11234723	[1]
LM095-11	*Citrus* x *aurantiifolia*	Rodrigues	2015	JAABII000000000	SRR11234722	[1]

LM095-14	*Citrus* x *aurantiifolia*	Rodrigues	2015	JAABIJ000000000	SRR11234721	[1]
LM096-03	*Citrus* x *aurantiifolia*	Rodrigues	2015	JAABIK000000000	SRR11234720	[1]
LM096-08	*Citrus* x *aurantiifolia*	Rodrigues	2015	JAABIL000000000	SRR11234719	[1]
LM096-09	*Citrus* x *aurantiifolia*	Rodrigues	2015	JAABIM000000000	SRR11234717	[1]
LM097-01	*Citrus* x *aurantiifolia*	Rodrigues	2015	JAABIN000000000	SRR11234716	[1]
LM121-01	*Citrus* x *limon*	Reunion	2015	JAABED000000000	SRR11234715	[1]

References

1. Richard D, Pruvost O, Balloux F, Boyer C, Rieux A, Lefeuvre P. Time-calibrated genomic evolution of a monomorphic bacterium during its establishment as an endemic crop pathogen. Mol Ecol. 2020;1–13. doi:10.1111/mec.15770

2. Gordon JL, Lefeuvre P, Escalon A, Barbe V, Cruveiller S, Gagnevin L, et al. Comparative genomics of 43 strains of *Xanthomonas citri* pv. *citri* reveals the evolutionary events giving rise to pathotypes with different host ranges. BMC Genomics. 2015;16:1–20. doi:10.1186/s12864-015-2310-x
