# Supplementary material for: First historical genome of a crop bacterial pathogen from herbarium specimen: Insights into citrus canker emergence
Source: PLoS Pathog. 2021 Jul 29;17(7):e1009714. doi: 10.1371/journal.ppat.1009714 (PMC8320980; doi:10.1371/journal.ppat.1009714)
Supplement: S2 Table — (PDF) [file ppat.1009714.s006.pdf]

S2 Table. List of *Xci* reference strain IAPAR 306 coding sequences (CDS) covered on less than 75% of their length by HERB_1937_Xci reads and hence designed as non-covered.

Sequence	CDS	Function	CDS start position	CDS end position	Coverage (%)	Probable cause of lack of coverage
Chromosome	*XAC0090*	ISxac3 transposase	107,220	107,492	0.00	Present in multiple copies
Chromosome	*XAC0091*	ISxac3 transposase	107,546	108,352	0.00	Present in multiple copies
Chromosome	*XANAC_0107*	Transposase fragment	108,217	108,387	0.00	Present in multiple copies
Chromosome	*XAC0093*	ISxac1 transposase	109,267	110,481	0.00	Present in multiple copies
Chromosome	*XANAC_0176*	Conserved protein of unknown function	177,476	177,610	44.44	-
Chromosome	*XAC0148*	ISxac1 transposase	179,328	180,542	1.32	Present in multiple copies
Chromosome	*XAC0152*	ISxac3 transposase	184,281	184,553	0.00	Present in multiple copies
Chromosome	*XAC0153*	ISxac3 transposase	184,607	185,425	3.05	Present in multiple copies
Chromosome	*XAC0342*	ISxac3 transposase	408,997	409,815	0.00	Present in multiple copies
Chromosome	*XAC0343*	ISxac3 transposase	409,869	410,141	0.00	Present in multiple copies
Chromosome	*XAC0502*	ISxac1 transposase	589,326	590,540	2.55	Present in multiple copies
Chromosome	*XAC0578*	ISxac3 transposase	681,530	682,348	0.00	Present in multiple copies
Chromosome	*XAC0579*	ISxac3 transposase	682,402	682,674	0.00	Present in multiple copies
Chromosome	*XAC0867*	Hypothetical protein	1,030,153	1,030,338	74.73	Present in multiple copies
Chromosome	*XAC0899*	Hypothetical protein	1,061,287	1,061,802	60.08	Present in multiple copies
Chromosome	*XAC0957*	Elongation factor Tu	1,116,063	1,117,253	45.59	Present in multiple copies
Chromosome	*XAC0970*	Elongation factor Tu	1,133,461	1,134,651	41.65	Present in multiple copies
Chromosome	*XANAC_1211*	Transposase fragment	1,210,215	1,210,385	17.54	Present in multiple copies
Chromosome	*XAC1052*	ISxac3 transposase	1,210,250	1,211,065	0.00	Present in multiple copies
Chromosome	*XAC1053*	ISxac3 transposase	1,211,110	1,211,382	0.37	Present in multiple copies
Chromosome	*XAC1070*	ISxac3 transposase	1,227,127	1,227,399	0.00	Present in multiple copies
Chromosome	*XAC1071*	ISxac3 transposase	1,227,453	1,228,271	1.83	Present in multiple copies
Chromosome	*XAC1102*	ISxac3 transposase	1,253,838	1,254,110	0.00	Present in multiple copies
Chromosome	*XAC1103*	ISxac3 transposase	1,254,164	1,254,970	0.00	Present in multiple copies
Chromosome	*XANAC_1273*	Transposase	1,254,751	1,255,005	0.00	Present in multiple copies

Chromosome	*XAC1440*	Hypothetical protein	1,661,955	1,662,686	49.86	Not present in all *Xci*
Chromosome	*XANAC_1729*	Protein of unknown function	1,729,998	1,730,132	74.07	Not present in all *Xci*
Chromosome	*XAC1504*	ISxcd1 transposase	1,736,814	1,737,080	0.00	Present in multiple copies
Chromosome	*XAC1505*	ISxcd1 transposase	1,737,074	1,737,901	0.00	Present in multiple copies
Chromosome	*XAC1508*	Hypothetical protein	1,741,181	1,742,077	57.75	Not present in all *Xci*
Chromosome	*XAC1613*	Hypothetical protein	1,859,901	1,860,470	73.68	-
Chromosome	*XAC1660*	ISxac3 transposase	1,912,359	1,912,631	3.30	Present in multiple copies
Chromosome	*XAC1661*	ISxac3 transposase	1,912,685	1,913,503	0.00	Present in multiple copies
Chromosome	*XAC1776*	Xylose isomerase 1	2,043,871	2,045,208	37.14	Present in multiple copies
Chromosome	*XAC1791*	Hypothetical protein	2,059,180	2,059,698	66.47	Not present in all *Xci*
Chromosome	*XAC1815*	Filamentous haemagglutinin	2,091,547	2,105,808	70.13	Fragment present in multiple copies
Chromosome	*XAC1816*	Haemagglutinin/hemolysin-related protein	2,106,435	2,110,727	69.46	Fragment present in multiple copies
Chromosome	*XANAC_2100*	Protein of unknown function	2,110,685	2,111,125	74.60	Not present in all *Xci*
Chromosome	*XAC1818*	Haemagglutinin	2,112,265	2,113,002	74.66	Fragment present in multiple copies
Chromosome	*XAC1916*	ISxac1 transposase	2,241,752	2,242,966	0.00	Present in multiple copies
Chromosome	*XAC1920*	ISxac3 transposase	2,246,692	2,247,498	0.00	Present in multiple copies
Chromosome	*XAC1921*	ISxac3 transposase	2,247,552	2,247,824	0.00	Present in multiple copies
Chromosome	*XAC1922*	Hypothetical protein	2,248,036	2,248,830	64.03	Not present in all *Xci*
Chromosome	*XAC1929*	ISxac1 transposase	2,253,897	2,255,111	7.24	Present in multiple copies
Chromosome	*XAC2099*	ISxac2 transposase	2,455,929	2,456,756	0.00	Present in multiple copies
Chromosome	*XAC2100*	ISxac2 transposase	2,456,750	2,457,016	0.00	Present in multiple copies
Chromosome	*XAC2101*	ISxac3 transposase	2,457,085	2,457,627	7.73	Present in multiple copies
Chromosome	*XAC2102*	ISxac3 transposase	2,457,681	2,457,953	0.00	Present in multiple copies
Chromosome	*XANAC_2465*	Protein of unknown function	2,486,685	2,487,209	61.14	Not present in all *Xci*
Chromosome	*XANAC_2469*	Protein of unknown function	2,488,298	2,488,552	5.10	Present in multiple copies
Chromosome	*XAC2131*	ISxac3 transposase	2,488,333	2,489,139	0.00	Present in multiple copies
Chromosome	*XAC2132*	ISxac3 transposase	2,489,193	2,489,465	0.00	Present in multiple copies
Chromosome	*XANAC_2524*	Transposase fragment	2,547,751	2,547,921	13.45	Present in multiple copies
Chromosome	*XAC2174*	ISxac3 transposase	2,547,786	2,548,592	0.00	Present in multiple copies
Chromosome	*XAC2175*	ISxac3 transposase	2,548,646	2,548,918	0.00	Present in multiple copies

Chromosome	*XAC2224*	ISxac3 transposase	2,608,668	2,608,940	0.73	Present in multiple copies
Chromosome	*XAC2225*	ISxac3 transposase	2,608,994	2,609,800	0.00	Present in multiple copies
Chromosome	*XANAC_2588*	Transposase fragment	2,609,665	2,609,835	0.00	Present in multiple copies
Chromosome	*XAC2371*	IS1479 transposase	2,764,543	2,764,899	32.21	Present in multiple copies
Chromosome	*XAC2424*	ISxcd1 transposase	2,826,253	2,827,080	0.00	Present in multiple copies
Chromosome	*XAC2426*	ISxcd1 transposase	2,827,074	2,827,340	0.00	Present in multiple copies
Chromosome	*XANAC_2944*	Putative secreted protein	2,967,575	2,967,907	74.17	Not present in all *Xci*
Chromosome	*XAC2604*	ISxac4 transposase	3,070,968	3,071,789	72.87	Present in multiple copies
Chromosome	*XAC2633*	ISxac3 transposase	3,096,744	3,097,016	0.00	Present in multiple copies
Chromosome	*XAC2634*	ISxac3 transposase	3,097,070	3,097,876	0.00	Present in multiple copies
Chromosome	*XANAC_3067*	Transposase fragment	3,097,741	3,097,911	15.79	Present in multiple copies
Chromosome	*XANAC_3074*	Conserved protein of unknown function	3,101,594	3,103,474	6.80	Present in multiple copies
Chromosome	*XANAC_3101*	Transposase fragment	3,119,621	3,119,791	0.00	Present in multiple copies
Chromosome	*XAC2661*	ISxac3 transposase	3,119,656	3,120,462	0.00	Present in multiple copies
Chromosome	*XAC2662*	ISxac3 transposase	3,120,516	3,120,788	0.00	Present in multiple copies
Chromosome	*XAC2860*	Hypothetical protein	3,355,194	3,357,104	7.54	Present in multiple copies
Chromosome	*XAC2889*	ISxac2 transposase	3,389,504	3,390,331	0.00	Present in multiple copies
Chromosome	*XAC2890*	ISxac2 transposase	3,390,325	3,390,591	0.00	Present in multiple copies
Chromosome	*XAC2898*	Type I restriction-modification system endonuclease	3,399,396	3,402,650	0.00	Not present in all *Xci*
Chromosome	*XAC2899*	Type I restriction-modification system specificity determinant	3,402,660	3,403,940	0.00	Not present in all *Xci*
Chromosome	*XAC2900*	Type I restriction-modification system DNA methylase	3,403,937	3,405,994	0.00	Not present in all *Xci*
Chromosome	*XAC2901*	Hypothetical protein	3,406,101	3,406,553	0.00	Not present in all *Xci*
Chromosome	*XAC2902*	Hypothetical protein	3,406,559	3,408,409	0.00	Not present in all *Xci*
Chromosome	*XAC2903*	Hypothetical protein	3,408,402	3,409,982	0.00	Not present in all *Xci*
Chromosome	*XAC2904*	Integrase/recombinase	3,409,969	3,411,288	0.00	Not present in all *Xci*
Chromosome	*XAC3221*	ISxac3 transposase	3,795,904	3,796,176	0.00	Present in multiple copies
Chromosome	*XAC3223*	ISxac3 transposase	3,796,230	3,797,036	0.00	Present in multiple copies
Chromosome	*XAC3247*	ISxac3 transposase	3,828,916	3,829,188	0.00	Present in multiple copies
Chromosome	*XAC3248*	ISxac3 transposase	3,829,242	3,830,060	0.00	Present in multiple copies
Chromosome	*XAC3282*	Integrase	3,863,304	3,864,302	16.22	Present in multiple copies

Chromosome	*XAC3283*	ISxac2 transposase	3,864,367	3,864,633	0.00	Present in multiple copies
Chromosome	*XAC3284*	ISxac2 transposase	3,864,627	3,865,454	0.00	Present in multiple copies
Chromosome	*XANAC_3872*	Transposase fragment	3,906,969	3,907,139	29.82	Present in multiple copies
Chromosome	*XAC3320*	ISxac3 transposase	3,907,004	3,907,810	1.98	Present in multiple copies
Chromosome	*XAC3321*	ISxac3 transposase	3,907,864	3,908,136	0.00	Present in multiple copies
Chromosome	*XAC3353*	Hypothetical protein	3,951,350	3,951,862	58.09	Present in multiple copies
Chromosome	*XAC3764*	ISxac2 transposase	4,441,928	4,442,194	0.00	Present in multiple copies
Chromosome	*XAC3765*	ISxac2 transposase	4,442,188	4,443,015	0.00	Present in multiple copies
Chromosome	*XAC3932*	Integrase/recombinase	4,624,144	4,625,418	0.00	Not present in all *Xci*
Chromosome	*XANAC_4559*	Protein of unknown function	4,626,191	4,627,177	0.00	Not present in all *Xci*
Chromosome	*XAC3933*	Hypothetical protein	4,627,179	4,629,134	0.00	Not present in all *Xci*
Chromosome	*XAC3934*	Hypothetical protein	4,629,124	4,629,558	0.00	Not present in all *Xci*
Chromosome	*XAC3935*	IS1389 transposase	4,629,977	4,630,213	0.00	Present in multiple copies
Chromosome	*XAC3936*	IS1389 transposase	4,630,220	4,630,882	0.00	Present in multiple copies
Chromosome	*XAC3937*	Hypothetical protein	4,631,131	4,631,694	0.00	Not present in all *Xci*
Chromosome	*XAC3938*	ISxac3 transposase	4,632,014	4,632,121	2.78	Present in multiple copies
Chromosome	*XAC3939*	ISxac2 transposase	4,632,230	4,632,355	0.00	Present in multiple copies
Chromosome	*XANAC_4568*	Protein of unknown function	4,632,628	4,632,810	0.00	Not present in all *Xci*
Chromosome	*XANAC_4569*	Protein of unknown function	4,632,751	4,632,930	0.00	Not present in all *Xci*
Chromosome	*XAC3940*	Hypothetical protein	4,632,933	4,634,114	0.00	Not present in all *Xci*
Chromosome	*XANAC_4572*	Protein of unknown function	4,634,408	4,634,626	0.00	Not present in all *Xci*
Chromosome	*XAC3941*	DNA helicase	4,634,759	4,636,588	0.00	Not present in all *Xci*
Chromosome	*XAC3942*	Hypothetical protein	4,636,552	4,638,234	1.25	Not present in all *Xci*
Chromosome	*XAC3943*	ISxac2 transposase	4,638,259	4,639,086	0.24	Present in multiple copies
Chromosome	*XAC3944*	ISxac2 transposase	4,639,080	4,639,346	0.00	Present in multiple copies
Chromosome	*XAC3945*	Recombination-like protein	4,639,395	4,639,580	0.00	Not present in all *Xci*
Chromosome	*XAC3946*	Hypothetical protein	4,639,840	4,640,757	0.00	Not present in all *Xci*
Chromosome	*XAC3947*	Hypothetical protein	4,641,520	4,642,101	0.00	Not present in all *Xci*
Chromosome	*XAC3948*	Hypothetical protein	4,642,098	4,643,435	0.00	Not present in all *Xci*
Chromosome	*XANAC_4582*	Protein of unknown function	4,643,652	4,643,858	0.00	Not present in all *Xci*

Chromosome	*XAC3949*	Hypothetical protein	4,643,991	4,644,626	0.00	Not present in all *Xci*
Chromosome	*XAC3950*	Hypothetical protein	4,644,639	4,645,520	0.00	Not present in all *Xci*
Chromosome	*XAC3951*	Hypothetical protein	4,645,856	4,646,824	0.00	Not present in all *Xci*
Chromosome	*XAC3952*	Hypothetical protein	4,646,821	4,649,073	0.00	Not present in all *Xci*
Chromosome	*XAC3953*	Hypothetical protein	4,649,598	4,649,912	0.00	Not present in all *Xci*
Chromosome	*XAC3954*	Hypothetical protein	4,649,962	4,650,735	0.00	Not present in all *Xci*
Chromosome	*XAC4094*	Membrane protein	4,799,656	4,799,883	0.00	Present in multiple copies
Chromosome	*XAC4095*	Hypothetical protein	4,799,873	4,800,100	0.00	Present in multiple copies
Chromosome	*XAC4137*	ISxac1 transposase	4,862,893	4,864,107	0.00	Present in multiple copies
Chromosome	*XAC4139*	Hypothetical protein	4,864,528	4,865,544	73.35	Not present in all *Xci*
Chromosome	*XAC4207*	ISxac3 transposase	4,956,111	4,956,383	0.00	Present in multiple copies
Chromosome	*XAC4208*	ISxac3 transposase	4,956,437	4,957,243	0.00	Present in multiple copies
Chromosome	*XAC4225*	Xylose isomerase 2	4,984,490	4,985,827	37.07	Present in multiple copies
Chromosome	*XAC4265*	Hypothetical protein	5,033,847	5,034,470	54.49	-
Chromosome	*XANAC_4980*	Protein of unknown function	5,071,090	5,071,275	35.48	Not present in all *Xci*
Chromosome	*XAC4322*	ISxac3 transposase	5,107,647	5,107,919	0.00	Present in multiple copies
Chromosome	*XAC4323*	ISxac3 transposase	5,107,973	5,108,779	0.00	Present in multiple copies
Chromosome	*XAC4325*	ISxac1 transposase	5,109,440	5,110,654	3.54	Present in multiple copies
Chromosome	*XAC4328*	ISxac1 transposase	5,116,871	5,118,085	0.00	Present in multiple copies
pXAC33	*XACa0022*	Avirulence protein (TAL)	13,079	16,459	37.27	Present in multiple copies
pXAC33	*XACa0039*	Avirulence protein (TAL)	27,299	30,589	10.94	Present in multiple copies
pXAC64	*XACb0015*	Avirulence protein (TAL)	14,558	17,848	12.09	Present in multiple copies
pXAC64	*XACb0064*	Hypothetical protein	54,556	55,008	8.61	-
pXAC64	*XACb0065*	Avirulence protein (TAL)	55,221	58,712	22.57	Present in multiple copies
