# Supplementary material for: First historical genome of a crop bacterial pathogen from herbarium specimen: Insights into citrus canker emergence
Source: PLoS Pathog. 2021 Jul 29;17(7):e1009714. doi: 10.1371/journal.ppat.1009714 (PMC8320980; doi:10.1371/journal.ppat.1009714)
Supplement: S3 Table — (PDF) [file ppat.1009714.s007.pdf]

S3 Table. List and coverage of 82 *Xanthomonas* virulence factors CDS (pthA4 not included) used in this study.

Gene family	CDS	Function	Coverage (%)	Found in modern *Xci* strains
avrBs2	*XAC0076*	Glycerophosphoryl diester phosphodiesterase	99.25	Yes
hpaA	*XAC0400*	Type III secretion control protein, maybe not a type III effector	100.00	Yes
hpaB	*XAC0396*	Type III secretion system chaperone	100.00	Yes
hpaC	*XAC0404*	Type III secretion system export control protein	100.00	Yes
hpaH	*XAC0417*	Type III secretion system putative transglycosylase HpaH	97.84	Yes
hpaI	*XANAC_475*	Type III effector HpaI protein (fragment)	100.00	Yes
hrcC	*XAC0415*	Type III secretion system outer membrane pore protein	100.00	Yes
hrcD	*XAC0399*	Type III secretion system protein	100.00	Yes
hrcJ	*XAC0409*	Type III secretion bridge between inner and outer membrane lipoprotein	100.00	Yes
hrcL	*XAC0411*	Type III secretion system cytoplasmic protein	100.00	Yes
hrcN	*XAC0412*	Type III secretion system ATP synthase	100.00	Yes
hrcQ	*XAC0403*	Type III secretion system apparatus protein	99.56	Yes
hrcR	*XAC0402*	Type III secretion system inner membrane protein	99.22	Yes
hrcS	*XAC0401*	Type III secretion system inner membrane protein	99.23	Yes
hrcT	*XAC0414*	Type III secretion system inner membrane protein	100.00	Yes
hrcU	*XAC0406*	Type III secretion system inner membrane protein	100.00	Yes
hrcV	*XAC0405*	Type III secretion system inner membrane channel protein	98.35	Yes
hrpB1	*XAC0407*	Type III secretion system protein	100.00	Yes
hrpB2	*XAC0408*	Type III secretion system protein	96.44	Yes
hrpB4	*XAC0410*	Type III secretion system protein	100.00	Yes
hrpB7	*XAC0413*	Type III secretion system protein	100.00	Yes
hrpD6	*XAC0398*	Type III secretion system regulator	96.30	Yes
hrpE	*XAC0397*	Type III secretion system pilin	100.00	Yes
hrpF	*XAC0394*	Type III secretion system translocator protein	98.42	Yes
hrpG	*XAC1265*	Type III secretion system OmpR-Type response regulator	94.44	Yes
hrpW	*XAC2922*	Pectate lyase, maybe not a Type III effector	99.34	Yes
hrpX	*XAC1266*	Type III secretion system transcriptional activator	100.00	Yes
xopA	*XAC0416*	Harpin, maybe not a Type III effector	96.86	Yes
xopAD	*XAC4213*	SKWP repeat protein	99.80	Yes
xopAE	*XAC0393*	LRR protein	100.00	Yes
xopAI	*XAC3230*	Putative ADP-ribosyltransferase	99.33	Yes
xopAK	*XAC3666*	Unknown	100.00	Yes
xopAP	*XAC2990*	Unknown	100.00	Yes
xopAQ	*XAC40v3*	Unknown	100.00	Yes
XopAU	*XAC1171*	Serine/threonine kinase	96.50	Yes
xopAY	*XAC1172*	Unknown	100.00	Yes
xopAW	*XAC2949*	Calcium-binding protein	99.40	Yes
xopAZ	*XAC1358*	SlpA superfamily, FKBP-Type peptidyl-prolyl cis-trans isomerase	99.58	Yes
xopC2	*XAC1210_Ψ*	Haloacid dehalogenase-like hydrolase (pseudogene)	100.00	Yes
xopE1	*XAC0286*	Putative transglutaminase	100.00	Yes
xopE2	*XACb0011*	Putative transglutaminase, plasmidic	100.00	Yes
xopE3	*XAC3224*	Putative transglutaminase	99.81	Yes
xopF1	*XANAC_0476_Ψ*	Unknown (pseudogene)	100.00	Yes
xopF1	*XANAC_0477_Ψ*	Unknown (pseudogene)	100.00	Yes
xopF2	*XAC2785_Ψ*	Unknown (pseudogene)	100.00	Yes
xopI	*XAC0754*	F-box protein	99.93	Yes
xopK	*XAC3085*	Unknown	99.67	Yes

xopL	*XAC3090*	LRR protein	100.00	Yes
xopM	*XAC0418*	Unknown	100.00	Yes
xopN	*XAC2786*	ARM/HEAT repeat	100.00	Yes
xopP	*XAC1208*	Unknown	95.48	Yes
xopQ	*XAC4333*	Putative inosine-uridine nucleoside N-ribohydrolase	100.00	Yes
xopR	*XAC0277*	Unknown	99.35	Yes
xopS	*XAC0315*	Unknown	98.6	Yes
xopV	*XAC0601*	Unknown	100.00	Yes
xopX	*XAC0543*	Unknown	100.00	Yes
xopZ1	*XAC2009*	Unknown	98.42	Yes
avrBs1	*XCVd0104*	Unknown	0.00	No
xopAA	*XCV3785*	Early chlorosis factor; Proteasome/cyclosome repeat	0.00	No
xopAF	*XOC_0445*	Unknown	0.00	No
xopAG	*XCC3600*	Unknown	5.08	No
xopAH	*XCC2109*	Unknown	0.00	No
xopAJ	*XCV4428*	Unknown	0.00	No
xopAL1	*XCC1246*	Unknown	0.00	No
xopAL2	*XCCB100_616*	Unknown	0.00	No
xopAM	*XCC1089*	Unknown	5.03	No
xopAX	*XCVd0086*	Unknown	0.00	No
xopB	*XCV0581*	Unknown	0.00	No
xopC1	*XCV2435*	Phosphoribosyl transferase domain and haloacid dehalogenase-like hydrolase	0.00	No
xopD	*XCV0437*	C48-family SUMO cysteine protease (Ulp1 protease family) (Clan CE); EAR motif; DNA binding; nuclear localization	0.00	No
xopF1	*XCV0414*	Unknown	0.00	No
xopF2	*XCV2942*	Unknown	6.84	No
xopG	*XCV1298*	M27-family peptidase (Clostridium toxin)	0.00	No
xopH1	*XCVd0105*	Putative tyrosine phosphatase	0.00	No
xopJ1	*XCV2156*	C55-family cysteine protease or Ser/Thr acetyltransferase (Clan CE)	0.00	No
xopJ3	*XCV0471*	C55-family cysteine protease or Ser/Thr acetyltransferase (Clan CE)	0.00	No
xopJ5	*XCC3731*	Putative C55-family cysteine protease or Ser/Thr acetyltransferase (Clan CE)	0.00	No
xopO	*XCV1055*	Unknown	0.00	No
xopT	*XOO2210*	Unknown	45.12	No
xopU	*PXO_00236*	Unknown	0.00	No
xopW	*PXO_03356*	Unknown	0.00	No
xopY	*XOO1488*	Unknown	0.00	No

Coding sequences (CDS) with name beginning by “XAC” or “XANAC” were predicted on the *Xci* reference strain IAPAR 306, while the other CDS were identified on other *Xanthomonas* strains (*Xanthomonas* resource website: http://www.xanthomonas.org/ [1]).

References

1. The *Xanthomonas* Resource (http://www.xanthomonas.org/t3e.html). 2018. Available from http://www.xanthomonas.org/t3e.html (accessed in May 2020)
