# Supplementary material for: First historical genome of a crop bacterial pathogen from herbarium specimen: Insights into citrus canker emergence
Source: PLoS Pathog. 2021 Jul 29;17(7):e1009714. doi: 10.1371/journal.ppat.1009714 (PMC8320980; doi:10.1371/journal.ppat.1009714)
Supplement: S4 Table — (PDF) [file ppat.1009714.s008.pdf]

S4 Table. List and frequency of nucleotide patterns coding for RVD found in HERB_1937 *Xci* reads.

RVD patterns	Occurrences (number of reads)	% of occurrences in HERB_1937	% of occurrences in modern strains	RVD	Possible RVD patterns of origin
CACGAT	25	35.71	29.77	HD	
AATATT	23	32.86	27.25	NI	
AATGGC	10	14.29	26.83	NG	
AAT---	8	11.43	9.01	N*	
AAA---	1	1.43	0	K*	AAT---
AATATC	1	1.43	0	NI	AATATT
CACGAA	1	1.43	0	HE	CACGAT
CAGGAT	1	1.43	0	QD	CACGAT
