# Supplementary material for: First historical genome of a crop bacterial pathogen from herbarium specimen: Insights into citrus canker emergence
Source: PLoS Pathog. 2021 Jul 29;17(7):e1009714. doi: 10.1371/journal.ppat.1009714 (PMC8320980; doi:10.1371/journal.ppat.1009714)
Supplement: S5 Table — (PDF) [file ppat.1009714.s009.pdf]

S5 Table. Description of the 14 SNPs found in coding regions between HERB_1937_Xci and modern strains of the SWIO clade.

Position	Name (codon & amino acid position)	Description on IAPAR 306 annotated genome	Amino acid		Change (score) Probable localization in protein
			HERB_ 1937_Xci	Modern SWIO strains	
581,148	XAC0494 (2, 398)	Two-component system sensor protein	Gly*	Glu[306]	non-conservative (-2)<br>no predicted domain
713,382	XAC0607 (2, 188)	Hypothetical protein with predicted transmembrane domain	Gly*	Asp[306]	non-conservative (-1)<br>adjacent to conserved domain of unknown function
922,664	XAC0778 (3, 56)	ftsW, cell division protein	Leu[306]	Leu[306]	synonymous
1,464,941	XAC1279 (2, 157)	Two-component system regulatory protein with histidine kinase domain	Arg*	Lys[306]	conservative (+2)<br>intracellular N-terminal domain [1]
3,116,890	XAC2657 (1, 15)	Peptidase S74 domain-containing protein	Ser[306]	Ala	conservative (+1)<br>variable a.a. in domain PHA02584
3,269,556	XAC2788 (3,39)	Hypothetical protein	Ala[306]	Ala[306]	synonymous
3,366,457	XAC2868 (3,279)	vieA, response regulator, diguanylate phosphodiesterase	Leu[306]	Leu	synonymous
3,529,273	XAC3022 (2, 186)	Unknown function	Thr[306]	Ile	non-conservative (-1)<br>in coiled domain
3,928,261	XAC3333 (3, 255)	Iron-regulated and PepSY-domain [2] containing protein of unknown function	STOP*	Gln[306]	truncated uncharacterised protein lacking 278/533 N-terminal a.a.
3,929,177	XAC3334 (2, 636)	fecA, Ton-B dependent receptor	Val*	Ala[306]	conservative (0)<br>intracellular β-sheet-20 [3]
3,958,174	XAC3361 (2, 28)	Hypothetical protein	Met	Thr[306]	non-conservative (-1)<br>+2 to predicted signal peptide cleavage site, possible effect on lipoprotein sorting [3]
4,388,772	XAC3712 (3, 264)	Metallopeptidase	Gln*	His[306]	conservative (0), in ectodomain. Unaffected catalytic signatures of Zn-binding M13 endopeptidases [4]
4,510,760	XAC3835 (2, 559)	icd, monomeric NADPH-dependent-isocitrate dehydrogenase	Glu[306]	Gly	non-conservative (-2)<br>cytoplasmic, α-helix next to hinge and binding domain
4,941,018	XAC4196 (1, 311)	YicJ, Na^+/melibiose symporter	Phe*	Leu[306]	conservative (0)<br>9th transmembrane domain, lacks conserved residues [5]

Amino acid substitutions between HERB_1937_Xci chromosome sequence and the sequences of 116 modern SWIO strains were analyzed using MaGe genome browser on *Xci* reference strain IAPAR 306 chromosome, and typical BLOSUM62 matrix scores [10]. When applicable, positions of non-synonymous mutations were deduced using dedicated references. For each SNP, amino acids found in HERB_1937_Xci only (*) were identified using BLASTp alignments of all non-redundant sequences (*Xanthomonas* taxid). Amino acids encoded by the same trinucleotides as in the reference strain IAPAR 306 are indicated ([306]). The SNP from non-coding region is not displayed.

References

1. Tomomori C, Tanaka T, Dutta R, Park H, Saha SK, Zhu Y, et al. Solution structure of the homodimeric core domain of *Escherichia coli* histidine kinase EnvZ. Nat Struct Biol. 1999;6:729–34. doi:https://doi.org/10.1038/11495

2. Yeats C, Rawlings ND, Bateman A. The PepSY domain: a regulator of peptidase activity in the microbial environment? Trends Biochem Sci. 2004;29:169–72. doi:10.1016/j.tibs.2004.02.004

3. Noinaj N, Guillier M, Barnard, TJ, Buchanan SK. TonB-dependent transporters: regulation, structure, and function. Annu Rev Microbiol. 2010;64:43–60. doi:10.1146/annurev.micro.112408.134247

4. Nagai S, Takada Y. Analysis of amino acid residues involved in the thermal properties of isocitrate dehydrogenases from a psychrophilic bacterium, *Colwellia maris*, and a psychrotrophic bacterium, *Pseudomonas psychrophila*. J Biosci Bioeng. 2020;129:284–90. doi:10.1016/j.jbiosc.2019.09.014

5. Yousef MS, Guan L. A 3D structure model of the melibiose permease of *Escherichia coli* represents a distinctive fold for Na+ symporters. PNAS. 2009;106:15291–6. doi:10.1073/pnas.0905516106

6. Henikoff S, Henikoff JG. Amino acid substitution matrices from protein blocks. PNAS. 1992;89:10915–9. doi:10.1073/pnas.89.22.10915
