# Supplementary material for: First historical genome of a crop bacterial pathogen from herbarium specimen: Insights into citrus canker emergence
Source: PLoS Pathog. 2021 Jul 29;17(7):e1009714. doi: 10.1371/journal.ppat.1009714 (PMC8320980; doi:10.1371/journal.ppat.1009714)
Supplement: Attachment — Submitted filename: Answer to reviewer.pdf [file ppat.1009714.s011.pdf]

We thank both the editors and reviewer#3 for these constructive comments and questions about our manuscript "First historical genome of a crop bacterial pathogen from herbarium specimen: insights into citrus canker emergence". You will find below, in red, a detailed list of our responses to the review comments, and a description of the changes we have made in the manuscript (we indicated the line numbers which have been changed, corresponding to our manuscript file without tracked changes). As requested, two versions of the revised manuscript: one with tracked changes denoting where the text has been changed; the other a clean version (uploaded as the manuscript file), have been uploaded.

On behalf of all co-authors,
Yours sincerely,
Adrien Rieux (corresponding author)

Reviewer #3: I appreciate that the authors took the time to reprocess their data as merged, as this did have an effect on their coverage estimates. I'm slightly curious as to why the merged data resulted in a greater number of genomic regions with no coverage, as I would have thought that merging would result in a reduction of reads spanning only those positions that were covered via paired end analysis. Was the map quality metric altered between these two analyses? Certainly increasing the map quality could lead to this phenomenon. Typically map quality values of 30 or even 37 are used, not just removal of reads with map quality 0.

>>> Map quality filters were kept identical between the two analyses and cannot explain why the merged data resulted in a greater number of genomic regions with no coverage. Further investigations would be required to answer this question but at this stage we can only hypothesize that merged (and longer) reads are more likely to display mismatches with the reference sequence, hence decreasing mapping efficiency and reducing coverage estimates.

Regarding coverage thresholds for SNP calling, the authors have now used average depth as a criterion for inclusion in the analysis, citing a study (ref 1 of their response) as a demonstration of this approach. While this approach is acceptable in their case, it should not be considered a global criterion for ancient DNA work, where coverage is often highly variable and can be very low. It is customary to set a threshold independent of coverage such as 3X or better yet 5X for SNP calling with ancient data.

>>> We agree with the above comment.

I thank the authors for including their percent Xci DNA in bulk content (0.74%). I have not seen them disclose their sequencing depth anywhere in the manuscript, and that should be mentioned ideally in the main text. Their genomic reconstruction was performed from 1.6 million mapping reads, so by extension I assume their sequencing depth was ca. 220 million reads.

>>> A total of 220.9 million of reads were generated by sequencing HERB_1937, as stated line 126 of our revised manuscript.

I'm also curious about why the BLAST analysis assigned 1.2% of bulk DNA to Xci. I would have thought BLASTn would have returned fewer reads assigned to Xci compared to mapping,

since the mapping process is based on read similarity only. Was a filtering step applied before the mapping? A comment about this difference could be useful.

>>> The filtering step (based on sequence quality only) was exactly the same between the “mapping-only” (assigning 0.74% of bulk DNA to Xci) and the “metagenomic” (assigning 1.20% of bulk DNA to *Xci*) pipelines. Our “metagenomic” pipeline assigned a larger proportion of reads to *Xci* because it was based on a combination of both mapping and BLASTn approaches, as now stated more clearly lines 146-148 of our revised manuscript.

A description of the mapping process and statistics of the Xci mapping should be presented in the main text before the damage assessment, which is calculated based on the mapped reads.

>>> Our revised manuscript has been reorganized by shifting around the “Historical genome reconstruction & characterization” and the “Ancient DNA damage assessment” sections.

They should also be clear on line 146 that their patterns of degradation apply to reads mapping to the Xci reference genome as opposed to simply “degradation in HERB_1937” as is it currently stated.

>>> Done. We now specifically mention that patterns of degradations were computed from HERB_1937_Xci, term defined as the portion of HERB_1937 mapping to *Xci* reference genome (lines 144 & 171)

Why are the damage profiles now shown in both the main manuscript (for the chromosome) and the SI (for the chromosome and two plasmids)? Why not just show the current SI image in the main text?

>>> A new figure 5 displaying damage profiles for the chromosome and two plasmids is now presented in the main text.

I also caution the authors against making assumptions that damage alone will “itself prove the authenticity” (wording from response letter) of their data. If they were to evaluate the human reads and observe a similar damage pattern, the data would have to be explained by contamination. Authenticity is conferred through damage profile along with context of the finding. Branch shortening in the phylogeny can be a useful metric for authentication of ancient data, and this is shown in their supplementary ML tree. The authors may wish to mention that as well at some point in the MS to bolster their claim of authenticity, along with highlighting its ancestral phylogenetic position (currently on line 329 outside the context of authenticity).

>>> We now mention the “branch shortening” metric, shown in the supplementary ML tree but also in the root-to-tip regression plot (Fig 6b) as an additional evidence for HERB_1937_Xci authenticity (see lines 275 & 322). We however decided not to add the argument of “ancestral phylogenetic position” in this context, as we believe this does not represent a metric for ancient data authentication in itself.

They entertain the possibility on lines 303 – 304 of their data coming from in-lab contamination, but I don’t think this statement is needed in light of the accompanying authenticity data they present.

>>> True. We adjusted this statement, while illustrating how insightful performing DNA damage patterns analysis on non-target species (here *Methylobacterium*) might be (lines 311-315).

Reporting on the mapping reads in their negative controls is currently missing in the main manuscript and absolutely must be included: This should also help to rule out in-lab contamination.

>>> We analyzed the 73,635 reads generated from our negative sample control: 10 mg of a herbarium plant sample that is not a host to *Xci*, processed in parallel to the HERB_1937 sample (lines 454, 473 & 485-488). No *Citrus* or *Xci*-specific DNA fragments were found in our negative control, thus ruling out in-lab contamination as now stated line 130.

The resolution of the main text images I received is of poor quality, and it looks like the samples have 20% damage as opposed to 2% damage, which really shocked and confused me at first. Please make sure that high resolution figures accompany the manuscript in print.

>>> New high-resolution figures have been submitted along with the revised version of our manuscript.

In their table 1 (main MS), I assume “most extreme position” refers to the terminal position? I’ve not come across that terminology and I find it unintuitive. Also, it is not common for % damaged bases to be presented as an average over the terminal five nucleotides, as the authors have done here. % damage at the terminal 5’ and 3’ ends is sufficient and is more commonly reported.

>>> We now report % deamination damage at the terminal 5’ and 3’ ends only in the revised version of our manuscript. Averaged values over the terminal five nucleotides were used to perform statistical comparison of deamination patterns between plasmids and chromosomal DNA.

Is it worth mention that the reads mapping to Xci are shorter than average for bulk DNA content and those mapping to Methylobacterium are longer than average? Do the authors know of a biological reason for this difference? GC content perhaps? Cellular structure?

>>> We did not attempt to explore the reasons for which the size of all, *Xci*-specific and *Methylobacterium*-specific reads slightly differs but this may be an interesting topic to cover in future study. The GC contents of both *Xci* and *Methylobacterium* reference strains are similar (64.7% and 66%, respectively) and do not suggest an effect on the differential degradation observed. We agree that a differential cell wall structure, for each of both Gram stain negative proteobacteria, may potentially be involved in the rate of DNA degradation, without discussing this point in the manuscript.
