# Supplementary material for: First historical genome of a crop bacterial pathogen from herbarium specimen: Insights into citrus canker emergence
Source: PLoS Pathog. 2021 Jul 29;17(7):e1009714. doi: 10.1371/journal.ppat.1009714 (PMC8320980; doi:10.1371/journal.ppat.1009714)
Supplement: Attachment — Submitted filename: Answer_to_reviewers.docx [file ppat.1009714.s010.docx]

**Answer to reviewers for the manuscript by Campos et al. "First historical genome of a crop bacterial pathogen from herbarium specimen: insights into citrus canker emergence"**

Dear editors,

We thank both the editors and the reviewers for their constructive comments and questions about our manuscript "First historical genome of a crop bacterial pathogen from herbarium specimen: insights into citrus canker emergence".

In order to address reviewer #3 comment (line 137 below), we re-ran most analyses on a new dataset built after merging the reads. Although this did not affect the outcome and the conclusions of our study, many calculated values and gene lists were updated in the revised version of our manuscript.

You will find below, **in blue**, a detailed list of our responses to the review comments, and a description of the changes we have made in the manuscript (we indicated the line numbers which have been changed, corresponding to our manuscript file without tracked changes).

As requested, two versions of the revised manuscript: one with tracked changes denoting where the text has been changed; the other a clean version (uploaded as the manuscript file), have been uploaded.

On behalf of all co-authors, yours sincerely,

Adrien Rieux, corresponding author

**Reviewer's Responses to Questions**

**Part I – Summary**

**Please use this section to discuss strengths/weaknesses of study, novelty/significance, general execution and scholarship.**

**Reviewer #1:** The manuscript entitled “First historical genome of a crop bacterial pathogen from herbarium specimen: insights into citrus canker emergence” by P.E. Campos and co-authors reports for the first time the description of the genome of a plant bacterial pathogen from a herbarium specimen. The authors targeted a 1937 specimen of Citrus sp. from Mauritius island (located in South West Indian Ocean, SWIO) showing typical symptoms of Asiatic citrus canker, an economically important disease caused by Xanthomonas citri pv. citri (Xci). They first confirmed the historical nature of the reconstructed genome HERB_1937 Xci by characterizing the DNA degradation patterns specific to ancient DNA. The authors further described the associated metagenome and again excluded recent contaminations by looking at the aDNA patterns of the most predominant taxon of the associated bacterial community. They investigated the HERB_1937_Xci gene content both on the chromosome and the two plasmids focusing on virulence factors and revealed a full equipment for virulence and detected three new allele sequence in TALE genes that are important for virulence. Then the authors compared the HERB_1937_Xci genome with a large set of modern genomes, especially including many genomes originating from the SWIO region. They performed a phylogenetic reconstruction which confidently placed HERB_1937_Xci at the root of the modern SWIO strains lineages making him a very likely member of the founder population. Having ensured the presence of a temporal signal, the authors finally estimated evolutionary parameters using a tip-calibration inference/ Bayesian tip-calibration inferences and refined the estimation of the age of the ancestor of modern SWIO strains proposing a scenario for the emergence of ACC in the SWIO region.

The authors have taken the opportunity of an immense resource of biodiversity that lies in the herbaria to increase our present knowledge on genetic diversity of a bacterial plant quarantine pathogen that still threatens large areas of citrus production. They reconstructed the introduction routes of this invasive bacterial species in SWIO. The evolutionary history that can be deduced provides information about the origin and history of the ACC emergence and the construction of the genetic diversity of the SWIO Xci populations.

They have produced original and new results that underline the interest of the methodological approach. From what I can judge, this methodological approach and the analyses implemented are appropriate and relevant to reach the conclusions presented. The manuscript is well written and the results are clearly presented.

**Reviewer #2:**

Strength: First study to sequence the whole bacterial genome of an important plant pathogen in a historic herbarium sample. Data analysis was thorough and paper is well- written

Weakness: Could have sequenced more specimens to address migration and root tree and examine evolution for genome over time. Did not justify why they chose this particular sample.

>>> We focused on one single specimen as we developed this study as a proof of concept on how to generate and analyze a crop bacterial pathogen historical genome from herbarium material and what conclusions can be drawn from such data. This particular sample (HERB_1937) was chosen as the oldest available symptomatic specimen from the SWIO area in the herbaria we have visited, as now stated in the revised version of our manuscript (lines 122 & 434).

**Reviewer #3:** This manuscript makes another contribution to a relatively underexplored area in ancient DNA – herbarium specimens from historical collections that show indications of infection. I was asked to comment specifically on the measures of authenticity regarding ancient DNA acquisition and analysis, so my comments largely relate to that. These comments aside, the paper is very clearly written and the analyses are extensive. I include a few comments below that might improve quality of the analysis.

**Part II – Major Issues: Key Experiments Required for Acceptance**

**Reviewer #1:** No major issues to be discussed here.

**Reviewer #2:**

• Need to sequence more historic genomes to justify some of their conclusions. That would make the paper stronger. They included a lot of previously sequenced genome data from modern lineages. For this work, I would ask them to qualify their conclusions and not do additional experiments

>>> We have now qualified some of our conclusions, stating the need to include more historical genomes in future work to strengthen them (lines 333 & 419).

**Reviewer #3:**

• First, it is typical in ancient DNA studies to eliminate all reads 30bp and shorter, after trimming (if this is done, which it is here). It seems the authors have not done so, despite their caution against it (line 172).

>>> Reads shorter than 30 nt were filtered-out after trimming (using Trimmomatic option “MINLEN:30”), as described line 469). For clarity, we removed “short” from the sentence line 171.

• The damage plot they present in Figure 4b is also confusing. The use of two horizontal axes, one on the top and one on the bottom, is confusing, as they are clearly independent, though at first glance they look as though they are describing the damage profiles of all reads 25bp in length. A more intuitive way to show their damage profiles would be to show the 5’and 3’ plots side by side. They could show only the first 25bp of the read in either direction, but this removes the ambiguity of the relationship between the positions in the 5’ and 3’ ends as shown in the current plot.

>>> Both figures 4b and S1b were redrawn accordingly, with only one horizontal axis and the 5’ and 3’ plots side by side.

• Line 61: Change “fossil remains” to “preserved tissues” or something similar, as fossils are rock with no preserved biological tissues

>>> Done.

• Line 105 – a sentence or two describing the preservation conditions of the sample would be helpful

>>> The Material & Methods section was modified to provide detailed information about the sample preservation conditions (lines 437-440).

• Line 109 – it’s not clear if the reads were merged, or simply treated as regular mate pair data. Reads should be merged, as treatment as modern paired end data would lead to incorrect base calling due to high background DNA. Treatment of each read independently will result in SNP calls in duplicate reads (via a call in both the forward and reverse read) and will inflate SNP coverage, which could be an issue with high non-target background. I suggest they merge their reads and reprocess the data.

>>> Done. Reads were initially treated as regular mate pairs. We have now merged the reads (as stated line 469) and totally reprocessed the data. This had downstream consequences, as summarized below:

- As expected, the mean depths of the reconstructed genomes were reduced by ～2x. They are now equal to 5.9, 21.9 and 17.3 for the chromosome, pXAC33 and pXAC64, respectively (updated in table 1 and figure 5)
- The number of genes considered to be absent in our historical genome has increased (139 instead of 80, see line 189).
- For SNP filtering, instead of using the previous arbitrary threshold of 15-fold depth, we now consider a metric proportional to the average depth of the reconstructed genome, as introduced in a previous study [1]. SNPs were now considered as dubious and filtered out when their depth<X, with X=average sequence depth + 1 sd (X=9 for HERB_1937_Xci). This is now detailed line 498.
- The total high-quality SNPs with the IAPAR 306 references slightly changed (83 instead of 81 for the chromosome, 4 instead of 10 for pXAC64, unchanged for pXAC33, updated line 241), with 78% of the new SNPs already present in the previously analyzed dataset.
- Analyzing the new set of SNPs between HERB_1937_Xci and its related SWIO clade, we obtained new, but very similar estimates of mean substitution rate and root age (see new values lines 278-282, figure 6 and figure S5). Importantly, we observed no effect on the reconstructed topology. Presence of genes coding for virulence factors and functional SNP characterization at the amino-acid level led to similar conclusions as well (lines 208-210, 376-384, 399-403).

• Line 116 – Greater clarity is needed here to explore how these percentages were generated. I assume via BLAST? What seems to be missing is a description of the percent Xci DNA in bulk DNA content based on mapping to a reference. This is a key value in aDNA studies.

>>> The metagenomic composition of HERB_1937 was estimated following a two-step procedure combining both reference-mapping and BLAST approaches, as described lines 472-480. In brief, reads were first mapped to human, *Citrus* and *Xci* reference genomes. In a second step, a subset of randomly chosen unmapped reads was blasted (using blast-n) on NCBI. The percentages on figure 3 were obtained by summing and scaling the number of reads assigned to the several taxa using the two approaches.

>>> 0.74 % of the total reads mapped to the *Xci* reference. This is indeed a key value that we have now included in the revised version of our manuscript (line 181 & Table 1).

• Line 127 – Remove or edit the passage “as compared to the standard read length of 150 nt obtained for modern Xci samples”, since read length is dependent on the sequencing platform and kit, and that is not clear based on this description.

>>> We removed this part.

• Line 144 – 148: I’m not sure why the authors opted to make this comparison. A far more informative metric would be to compare the Xci DNA damage profile to that of the host (perhaps to a plastid or a chromosome). That is typically done as a means of authenticity in ancient pathogen investigations.

>>> We made this comparison because *Methylobacterium* has been identified as a major lab contaminant in modern sample sequences [2], as stated line 304. By analyzing its damage patterns, our results suggest its presence in HERB_1937 was historical (more or less contemporary with the *Xci* infection) rather than caused by a more recent laboratory contamination. We did not aim to analyze *Citrus* DNA damage profile for two main reasons. First, *Xci* DNA degradation patterns itself prove the authenticity of our reconstructed genome. Second, as the host plant was not confidently identified to the species level, deamination estimations could suffer from the lack of a sufficiently closely related reference sequence, as described previously [3].

• Table 1: Column 2 should be % of reads mapping to the reference based on total DNA content (% endogenous DNA), before duplicate removal. Currently this represents the proportion of mapped reads that assigns to the genetic component, which isn’t as informative. Column 3 is called “Mean depth”, though I suppose they mean “Mean coverage”, which is a clearer term. Last column, which shows the % reads with damaged terminal bases show values between 1.62 and 1.88, though 2.64% is reported in the main text (line 135). Why the discrepancy?

>>> Column 2 now indicates the % endogenous DNA content before duplicate removal.

>>> In column 3, we prefer to use the term “mean depth”, and use “coverage” in column 4 to describe the percentage of reference sequence covered by a given depth. We now provide a clear definition to avoid any misunderstanding (see Table 1 & line 182).

>>> The last column gives a mean value calculated on the 5 extreme nucleotides on both 5’ and 3’ ends, while 2.64% corresponds to the value of the most extreme (and unique) position as stated lines 154-155 & 167, respectively.

• Lines 168 – 169: Reporting % of the reference covered at 1X is not terribly informative, especially if base calling required a min of 15-fold coverage (as is described in the methods). Also is it not clear if the map quality was 0 or higher. If it was 0, the authors are reporting reads that will map to multiple locations in the genome, which could inflate their coverage, especially if these positions are later removed for SNP calling.

>>> We believe that “mapping” and “variant calling” -associated statistics should be given independently as they both provide different but useful insights. The percentage of the reference covered at 1X illustrates to which extent the genomic composition of the reference genome is shared by the reconstructed historical genome, independently of the threshold chosen for a SNP to meet our quality filters.

>>> We did not filter for mapping quality when computing those statistics; mapping was performed with BWA-aln 0.7.15 (default options and seed disabled), which randomly places repetitive reads (tagged with a mapping quality of 0) rather than mapping them to multiple locations. To verify the consistency of our results, we also computed coverage values after excluding reads with a mapQ=0 and obtained extremely similar values (maximum difference observed for plasmid pXAC33 coverage at 5X: 80.0 instead of 80.2%).

• Line 273 – “obtained from such material”… greater specificity is needed here to describe what “such material” is.

>>> Done, “such material” has been replaced by “herbarium material” (line 290).

• Line 277 – “contamination-free environment”: no environment is contamination-free.

>>> This element has been removed from this section (line 294).

• Lines 291 – 293: There is no need to explore the reasons for 60% of the reads not mapping to a reference. This is a nearly ubiquitous phenomenon in ancient DNA work, and probably also in modern metagenomics studies.

>>> Those reads are actually both not mapping to a reference, nor being assigned with BLAST. We do not explore further the underlying reasons but now acknowledge the ubiquity of such an observation (line 312).

• Line 295 – change “studies” to “study”

>>> Done.

• Line 297 – were the preservation conditions for P. infestans similar?

>>> This is hard to know and we did not discuss this point further as all herbaria are potentially exposed to different preservation conditions that are, to our knowledge, rarely recorded.

• Line 298 – “in the reads mapping to either of two plasmids” is a better way to say this

>>> Done.

• Lines 331 – 334: If a conclusion is being drawn from tree topology, the ML tree with bootstraps should ideally be included in the main text. Currently the authors include only the Bayesian tree, which was constructed with the ML tree as a prior (if I have understood the methods properly)

>>> The Bayesian tree was not constructed using the ML tree as a prior. We now clarify the methods section (line 549). We computed a ML tree (without constraining tip dates) to test the presence of temporal signal, as required to perform Bayesian tip-calibrated inferences. We also aimed to compare the ML and the Bayesian topologies, as incongruences between them can erroneously affect Bayesian evolutionary estimates [4]. As our ML and Bayesian trees were congruent (similar topology and node supports), we decided to include only the latter in the main text as it is also time-calibrated.

• Line 361 – typo “deshydrogenase”

>>> Corrected to “dehydrogenase” (new line 381)

• Lines 392 – 403: There is some odd grammar in this concluding paragraph, and the English should be adjusted for better clarity

>>> We accordingly rephrased our concluding paragraph.

**Part III – Minor Issues: Editorial and Data Presentation Modifications**

Please use this section for editorial suggestions as well as relatively minor modifications of existing data that would enhance clarity.

**Reviewer #1:** Minor comments are indicated below

The first three points are free comments and do not necessarily need to be followed up on.

• Line 45. And to possibly close the loop, loss of biodiversity can impact disease risk (Keesing, 2006, Ecol. Lett. 9:485; Rohr et al., 2020, Nature Ecol. & Evol. 4:24).

• Maybe you could refer to the pioneering work of Ristaino et al (2001, Nature 411:695).

>>> Done (line 80, reference 15).

• Line 91. You could add a sentence highlighting further the contribution of herbarium specimens in deciphering the original outbreak of ACC in Florida at the beginning of the twentieth century (Li et al., 2007).

>>> Done (lines 106-108).

• Line 105 (and line 416). DNA was extracted from a pool of five lesions which could represent independent infection events. Is there any possibility of strains mixture? Or did you observe any sequence variation within HERB_1937_Xci that could be attributed to the presence of different strains?

>>> In a recent study, Pruvost *et al.* [5] showed that strains-mixture due to polyclonal infection may exist within single *Xci* lesions. As such, pooling five lesions from the same leaf could have resulted in HERB_1937_Xci being reconstructed from a mixture of strains. We investigated this phenomenon and observed rare traces of within-sites heterogeneity in HERB_1937_Xci. However, we could not confidently attribute the variants to distinct strains, nor rule out the possibility of them arising from other causes, such as bad mapping. Hence, we labelled such sites as dubious and masked them from genome reconstruction and phylogenetic inferences.

• Line 115. You talk about identified sequences at the species level but the taxonomic level of the plant host is at the genus level. Even if this was not your objective, did you get any clues from the reads about the species identification of your citrus specimen?

>>> We accordingly qualified *Citrus* sequence identifications at a genus level (line 136). During the metagenomic BLAST analysis, reads were assigned to seven *Citrus* species (*C. aurantiifolia, C. cavaleriei, C. clementina, C. depressa, C. limon, C. medica and C. sinensis*) with almost 90% of them assigned to *C. sinensis*. However, only nine complete *Citrus* genomes are present in NCBI database, which does not allow for a representation of many hybrids and varieties, and therefore can skew HERB_1937 identification to *Citrus* species level. For this reason we decided not to discuss further this result in our manuscript.

• Line 199. Change to S3 Table for homogenization.

>>> Done.

• Line 261. Check with Fig 6b where R2 = 0.043 and p-value = 0.0105.

>>> Checked and corrected. Note that consistent new values (line 277) are now given in the revised version of our manuscript, as we reanalyzed a new dataset built by merging the reads (see answer to third reviewer).

• Line 312. Did you estimate any genetic diversity indices to support this assertion?

>>> This assertion was previously based on the visual observation that Mauritian strains were present in all lineages of the phylogeny. We have now computed phylogenetic diversity indices to support our observation (lines 270 & 559).

• Line 336. Can a privileged ancient history between these two islands explain older migration events and shed light on the presence of older Xci common ancestors?

>>> Indeed, Reunion and Mauritius islands were politically connected during the 18th century and early 19th century (both were French colonies), with many described exchanges of goods and working people. This has now been stated line 356.

Bibliography

• Line 645. Change to canker-like.

>>> Done.

• Line 743. Reference Bolger et al. to be completed.

>>> Done.

• Line 776. Prefer: Jombart T., Balloux F., Dray S., adephylo: new tools for investigating the phylogenetic signal in biological traits, Bioinformatics, Volume 26, Issue 15, 1 August 2010, Pages 1907–1909, https://doi.org/10.1093/bioinformatics/btq292

>>> Done.

• I could not consult the data associated with HERB_1937 deposited to the SRA and on GenBank database which were not yet available.

>>> The data deposited on NCBI will only be publicly available after publication acceptance. For the SRA (raw reads), we managed to request a private link giving you access to the data before public release:

<https://dataview.ncbi.nlm.nih.gov/object/PRJNA668001?reviewer=k1jv3cmf9996v13ditoh5plodj>

However, this was not possible for the reconstructed genomes. For you to be able to consult those files, we have deposited them, along with a screenshot of the GenBank website, on the following webpage: <https://adrienrieux.wordpress.com/shared-data/>

**Reviewer #2:** Page Line Comments

• Abstract 1 Change to “ancient genomics has been used”

>>> Done.

• 43 Add a . after (3) and start new sentence with “More”

>>> Done (line 57), starting new sentence with “Simultaneously”.

• 46 Change to “infectious crop” diseases

>>> Done.

• 54 Rework this sentence as there have been studies with herbarium collections that sampled over more than 4 decades. See paper by Ristaino et al “Ristaino, J. B. 2020. The importance of mycological and plant herbaria in tracking a plant killer. Front. Ecol. Evol. 7:521. doi: 10.3389/fevo.2019.00521” for a review of the studies with plant mycological collections to track plant disease epidemic”.

>>> The sentence was modified (line 70) to refer to studies performed on modern field samples only. We now cite the paper by Ristaino [6] suggested above in the following section introducing the role of herbarium collections in understanding the evolution and epidemiology of plant pathogens (line 96).

• 58 Delete “to” and change to “systemic detection of evolutionary changes or reconstruction of …”

>>> Done (line 73). Our initial sentence referred to a thorough, rather than to a systemic detection; the sentence was changed accordingly.

• 63 Add more citations here. Yoshida was not the first to use herbarium specimens to identify the famine-era lineage of P. infestans. Cite “May, K. J. and Ristaino, J. B. 2004. Identity of the Mitochondrial DNA Haplotype(s) of Phytophthora infestans in Historical Specimens from the Irish Potato Famine. Mycol. Res. 108:171-179.”

>>> Done (line 80).

• 81 Add more citations here “Saville, A. , Martin, M, Gilbert, T. and Ristaino, J. 2016. Historic late blight outbreaks caused by a widespread dominant lineage of Phytophthora infestans (Mont.) de Bary Plos One 11: e0168381 https://doi.org/10.1371/journal.pone.0168381” and others mentioned previously. I’d like to think our work has helped resolved the debate about the identity and source of the 19th century outbreak.

>>> References were added (line 80).

• 84 Cite Li et al. (26) here as Hartungs work did use herbarum specimens to study citrus canker and I believe did the first genetic work with a historic strain from 1911. Although they did not use full genomes sequences they did to multilocus genotyping so say a bit more here and compare your results to previous work in the discussion.

>>> Done (lines 107 & 277).

• 104 Indicate the name of the herbarium that the sample was retrieved from and define SWIO.

>>> Done (see line 121). SWIO (South West Indian Ocean) is defined earlier in the manuscript (lines 109).

• 116 There was a greater percentage of reads from some other bacterial species than X. citir (1.2% vis Methylbacterium 1.57%) Methylobacterium has been identified as a contaminant during DNA extraction and can lead to its erroneous appearance in microbiota or metagenomic datasets. Did you run a preliminary PCR on the samples to confirm they were actually infected with X citri before the llumina sequencing using species specific primers. Rep or Box PCR, etc It would be good to conform or look for sequences specific to the pathogen to confirm that the samples were infected by X citri.

>>> The *Methylobacterium* genus has been described as a prominent core-member of the microbiota of citrus leaves, with a relative abundance among bacteria (from 5 to 58% [7]); consistent with our data (11,7%). We measured the levels of DNA degradation for *Methylobacterium* sequences, which suggested it is an actual component of the herbarium specimen microbiota rather than a (modern) lab contaminant. We therefore concluded that the *Methylobacterium* genus may be representative of the endogenous microbiota of the *Citrus* leaf used in our study (reviewed in [8]) as now discussed lines 304 to 307).

>>> For *Xanthomonas*, we used a real-time PCR diagnostic assay [9], whose target sequence (included in CDS XAC1051) ensures a specific and exclusive amplification of *Xci* (*i.e.*, no amplification of other *Xanthomonas* species), to confirm that HERB_1937 was infected by *Xci* prior to sequencing. This has now been included in the revised version of our manuscript (lines 126 & 454).

• 122 Could reduce some of this section about DNA damage as its clear you are using herbarium material and the read will likely be short.

>>> There is only one sentence about fragment length in this section. Although reads were indeed expected to be short, it is important to report their size distribution as it is a proof of authenticity for our historical genome.

• 175 Did you sequence more than one herbarium sample? Why was this particular sample of interest. Is the oldest existing specimen? It would have been better to sequence more samples from the various islands and at different times in the past to present day.

>>> We only sequenced this herbarium sample during the course of this study. We chose it as it was the oldest *Xci*-infected *Citrus* specimen we could find from the SWIO area, as stated line 434. More historical genomes from different islands of the area and from various ages will be interesting to generate and analyze in a future project. Note that the “modern” genomes we included in our analysis were generated from strains sampled in the field between 1978-2015 and represent as such different time-points between HERB_1937 and the most recent (2015) isolates (Table S1).

• 199 The data on the T3SS is interesting. Were the absent genes viewed necessary for virulence in modern lineages?

>>> All 25 absent CDS were identified in *Xanthomonas* not *Xci* and are not necessary for *Xci* pathogenicity, thus not expected to be present. We now describe explicitly where these 25 CDS were from (line 211).

• 215 This mapping method that uncovered new reads in the TALE region is interesting

• 227 Say more about the number and location of the SNPS. What do you mean by “dubious SNPS”?

>>> This section describes the SNPs observed between the historical genome HERB_1937_Xci and the IAPAR 306 reference genome. We found a total of 88 SNPs, among which 83 were on the chromosome, 4 on plasmid pXAC64 and 1 on plasmid pXAC33. We decided not to describe their location (the annotation of their genetic support) any further, as we performed a more meaningful analysis for SNPs identified between HERB_1937_Xci and its related SWIO clade (see line 252).

>>> Dubious SNPs are SNPs filtered out because of a low depth, SNP heteroplasy or proximity with another variant, as now defined more clearly lines 240 & 499.

• 268 I can’t help but think that sequencing more samples would also have improved the root date of the tree. What do you know about movement of citrus during this time period? Are there historical records on the introduction of the pathogen that might indicate times of introduction?

>>> Looking at the influence of our historical genome on the inference of root age, one might hypothesize that adding more historical samples might our estimation. Only future studies will allow testing it, as acknowledged in the revised version of our manuscript (line 333).

>>> As stated line 340, we could find two major historical events associated with citrus movement in the SWIO area during this time-period: (1) the travels between Asia and SWIO islands of the botanist Pierre Poivre and (2) the movement of indentured laborers from Asia to French and British colonies after the abolishment of slavery to work in agriculture.

>>> The oldest historical record of ACC disease in the area was made in Mauritius in 1917 (line 336) which is in accordance with the age of the ancestor of all SWIO strains we estimated to be at 1843 [95% HPD: 1803 – 1881].

• 274 Add more citations here as stated above on page 3 line 54.

>>> In this section, we referred to studies in which whole genomes of pathogens were reconstructed from herbarium material. Other studies that focused on a subset of genes or targeted specific genetic markers were cited elsewhere (lines 80 & 108).

• 284 Sequencing more samples would have given you an indication of whether this was contamination or infection by other bacteria.

>>> We discuss this point (lines 299-307) in light of the recent work of Bieker *et al.* (2020) [10] demonstrating that it is possible to use herbarium specimens for metagenomic analyses but that caution should be taken when analyzing data as some of the identified species may be herbarium contaminants rather than representing the natural metagenomic community of the host plant. Noteworthily, the three most prominent genera we detected (*Methylobacterium*, *Curtobacterium* and *Sphingomonas*, >0.5% of aligned reads) belong to the five genera of the core citrus leaf microbiome (reviewed in [8]). We thus hypothesized that these genera were likely associated to the living citrus plant, adding this information to the discussion (line 307).

• 320 Are the French botanists samples available in the Paris herbarium? These would be interesting to see if you can push back the introduction date.

>>> Unfortunately, we could not find any *Citrus* samples collected by Pierre Poivre in collection at the Paris herbarium.

• 331 You really need more samples to determine if one or more introductions occurred or even it the same pathovar was introduced. This is speculation so reword this.

>>> We have reworded accordingly (line 351). The other pathovars targeting citrus are *X. citri* pv. *bilvae* (*Xcb*) and *X. citri* pv. *aurantifolia* (*Xca*). The hypothesis of an introduction with one of those two pathovars appears unlikely as *i*) *Xcb* induces fairly different symptoms and has never been described in the SWIO and *ii*) *Xca* has so far only been observed in South America.

• 333 You could do migration analysis on the data to see if directional gene flow has occurred among the island nations.

>>> We decided not to perform such migration analysis for the purpose of this study as we believe our data is not appropriate for two main reasons. First, the most common algorithms (IM, MIGRATE or DaDi) assume unlinked variant sites which conflict with the clonal reproduction of *Xci*. Second, such inferences require random and homogeneous sampling strategies of populations, which is not the case in our dataset.

• 363 How would this influence pathogenicity – more or less pathogenic? Is the historic lineage potentially more virulent then modern day lineages or not?

>>> SNPs were reassessed following a new analysis using merged reads (response reviewer #3 line 137), with 78% of the newly identified SNPs already present in the previous dataset. Among the now 14 described SNPs in coding regions, six are non-synonymous and non-conservative, with possible functional consequences. Assumptions could be made on one characterized gene, whose functional domains are well-described: *icd*, monomeric NADPH-dependent-isocitrate dehydrogenase (see our discussion, lines 379-381). This gene is essential for the tricarboxylic acid cycle (or Krebs cycle) of most bacteria. The mutation described in our paper may have functional consequences on the ability of the bacteria to proliferate. However, as for any mutation with potential functional consequences, this aspect will need to be tested *in vivo*, in an integrated context, using directed mutagenesis (for example, [11]).

• 374 I encourage you to continue more sequencing of historic genomes of this important pathogen so you can try de novo genome assembly and do further migration and phylogenetic analysis. Search for specimens in unlikely places. They may be in museum collections from explorers to the region including the British.

>>> *De novo* assembly would be particularly useful as it would allow detecting any genetic material present in historical genomes but absent in the reference sequences as stated line 395. However, the fragmented nature of nucleic acids, as well as the presence of repetitive elements (for example *Xci* contains almost 100 CDS predicted as transposases) are a serious handicap for *de novo* assembly.

**Reviewer #3:** (No Response)

**References**

1.	Richard D, Pruvost O, Balloux F, Boyer C, Rieux A, Lefeuvre P. Time‐calibrated genomic evolution of a monomorphic bacterium during its establishment as an endemic crop pathogen. Mol Ecol. 2020;1–13. doi:10.1111/mec.15770

2.	Glassing A, Dowd SE, Galandiuk S, Davis B, Chiodini RJ. Inherent bacterial DNA contamination of extraction and sequencing reagents may affect interpretation of microbiota in low bacterial biomass samples. Gut Pathog. 2016;8:1–12. doi:10.1186/s13099-016-0103-7

3.	Prüfer K, Stenzel U, Hofreiter M, Pääbo S, Kelso J, Green RE. Computational challenges in the analysis of ancient DNA. Genome Biol. 2010;11:1–15. doi:10.1186/gb-2010-11-5-r47

4.	Emerson BC, Alvarado-Serrano DF, Hickerson MJ. Comment on Rieux and Balloux: calibration from tip-dating can compromise topological accuracy and evolutionary inference. Mol Ecol. 2017;26:2623–4. doi:10.1111/mec.13951

5.	Pruvost O, Boyer K, Ravigné V, Richard D, Vernière C. Deciphering how plant pathogenic bacteria disperse and meet: Molecular epidemiology of *Xanthomonas citri* pv. *citri* at microgeographic scales in a tropical area of Asiatic citrus canker endemicity. Evol Appl. 2019;12:1523–38. doi:10.1111/eva.12788

6.	Ristaino JB. The importance of mycological and plant herbaria in tracking plant killers. Front Ecol Evol. 2020;7:1–11. doi:10.3389/fevo.2019.00521

7.	Blaustein RA, Lorca GL, Meyer JL, Gonzalez CF, Teplitski M. Defining the core citrus leaf- and root-associated microbiota: factors associated with community structure and implications for managing huanglongbing (citrus greening) disease. Appl Environ Microbiol. 2017;83:1–14. doi:10.1128/AEM.00210-17

8.	Zhang Y, Trivedi P, Xu J, Roper MC, Wang N. The citrus microbiome: from structure and function to microbiome engineering and beyond. Phytobiomes J. 2021;1–40. doi:10.1094/PBIOMES-11-20-0084-RVW

9.	Robène I, Maillot-Lebon V, Chabirand A, Moreau A, Becker N, Moumène A, et al. Development and comparative validation of genomic-driven PCR-based assays to detect *Xanthomonas citri* pv. *citri* in citrus plants. BMC Microbiol. 2020;20:1–13. doi:10.1186/s12866-020-01972-8

10.	Bieker VC, Sánchez Barreiro F, Rasmussen JA, Brunier M, Wales N, Martin MD. Metagenomic analysis of historical herbarium specimens reveals a postmortem microbial community. Mol Ecol Resour. 2020;1–14. doi:10.1111/1755-0998.13174

11.	Nagai S, Takada Y. Analysis of amino acid residues involved in the thermal properties of isocitrate dehydrogenases from a psychrophilic bacterium, *Colwellia maris*, and a psychrotrophic bacterium, *Pseudomonas psychrophila*. J Biosci Bioeng. 2020;129:284–90. doi:10.1016/j.jbiosc.2019.09.014
